# Vibrational Tunneling Spectra of Molecules with Asymmetric Wells: A Combined Vibrational Configuration Interaction and Instanton Approach

**DOI:** 10.1021/acs.jctc.2c00124

**Published:** 2022-04-19

**Authors:** Mihael Eraković, Marko T. Cvitaš

**Affiliations:** † Department of Physical Chemistry, Rud̵er Bošković Institute, Bijenička Cesta 54, 10000 Zagreb, Croatia; ‡ Department of Physics, Faculty of Science, University of Zagreb, Bijenička Cesta 32, 10000 Zagreb, Croatia

## Abstract

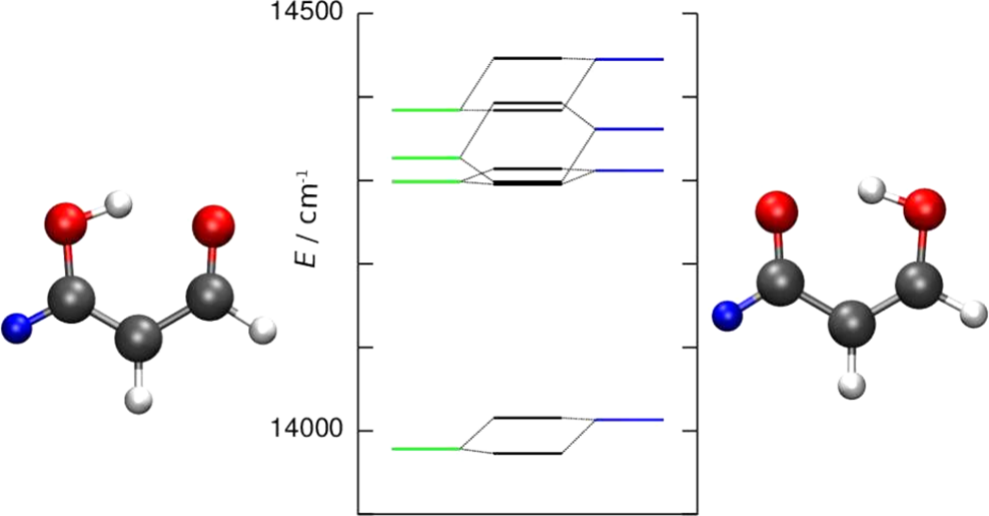

A combined approach that uses the vibrational configuration interaction (VCI) and semiclassical instanton theory was developed to study vibrational tunneling spectra of molecules with multiple wells in full dimensionality. The method can be applied to calculate low-lying vibrational states in the systems with an arbitrary number of minima, which are not necessarily equal in energy or shape. It was tested on a two-dimensional double-well model system and on malonaldehyde, and the calculations reproduced the exact quantum mechanical (QM) results with high accuracy. The method was subsequently applied to calculate the vibrational spectrum of the asymmetrically deuterated malonaldehyde with nondegenerate vibrational frequencies in the two wells. The spectrum is obtained at a cost of single-well VCI calculations used to calculate the local energies. The interactions between states of different wells are computed semiclassically using the instanton theory at a comparatively negligible computational cost. The method is particularly suited to systems in which the wells are separated by large potential barriers and tunneling splittings are small, for example, in some water clusters, when the exact QM methods come at a prohibitive computational cost.

## 1 Introduction

Physical systems with multiple energetically stable minima are ubiquitous in chemistry and physics.^1^ Bound states that are localized in such wells, separated by potential barriers, interact via quantum tunneling, which results in observable shifts of their energies.^2,3^ For equivalent, symmetry-related wells, the states that would be degenerate in the absence of tunneling produce a splitting pattern of energy levels.

Molecules and molecular complexes with two or more equivalent stable configurations are multidimensional systems that display these effects in their vibrational spectrum. The inversion of ammonia,^4^ proton tunneling in malonaldehyde,^5^ double proton transfer in porphycene,^6^ or bond rotation in the vinyl radical^7^ are examples of symmetric double-well systems that produce measurable tunneling splittings (TSs) of their vibrational state energies. Water clusters are prototype multiwell systems that exhibit nontrivial splitting patterns caused by tunneling rearrangements between many stable configurations of the cluster.^8^

The asymmetric systems, which have nonequivalent wells, have been less studied. When the state energies of different wells are in resonance, the tunneling dynamics will again cause the delocalization of the wavefunction across the wells and the energy shifts in the spectrum.^3^ Away from the resonance, the states remain localized in one well. The asymmetry can be induced in symmetric molecular systems by asymmetric isotopic substitutions.^9^ The normal modes and vibrational frequencies in equivalent symmetry-related potential wells then differ, and the correspondence of the vibrational wavefunctions of different wells is not preserved in general. As an example, the malonaldehyde molecule deuterated at the D7/D9 position (see Figure 4) thus has an asymmetric level structure with the localized states and those that are delocalized across the two minima.^9^ A mixing angle between the left–right ground vibrational states of partially deuterated (PD) malonaldehyde has been determined experimentally.^10^ Further examples of the mixing have been studied in the HF–DF dimer^11^ and PD vinyl radical,^12^ CHD–CH, using full-dimensional exact calculations. The bifurcation splitting patterns in PD water trimers HDO(H_2_O)_2_ and D_2_O(H_2_O)_2_ have been determined in experiments^13^ and by us using the instanton theory.^14^

**Figure 1:**
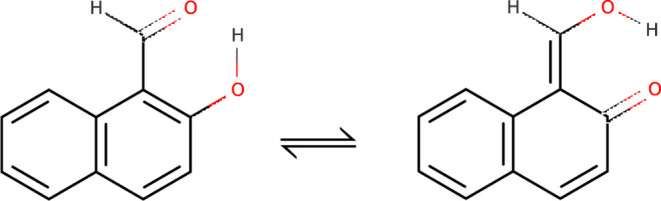
Tunneling tautomers of 2-hydroxy-1-naphthaldehyde.

**Figure 2:**
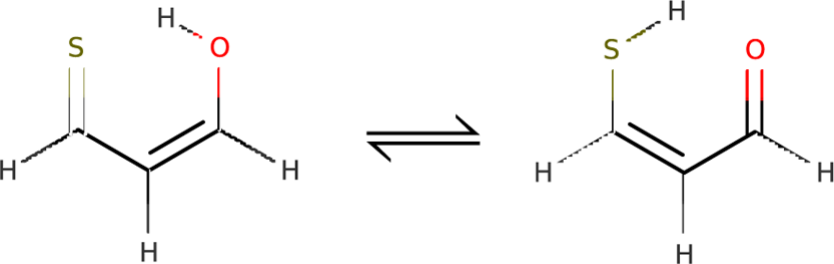
Tunneling tautomers of thiomalonaldehyde.

**Figure 3:**
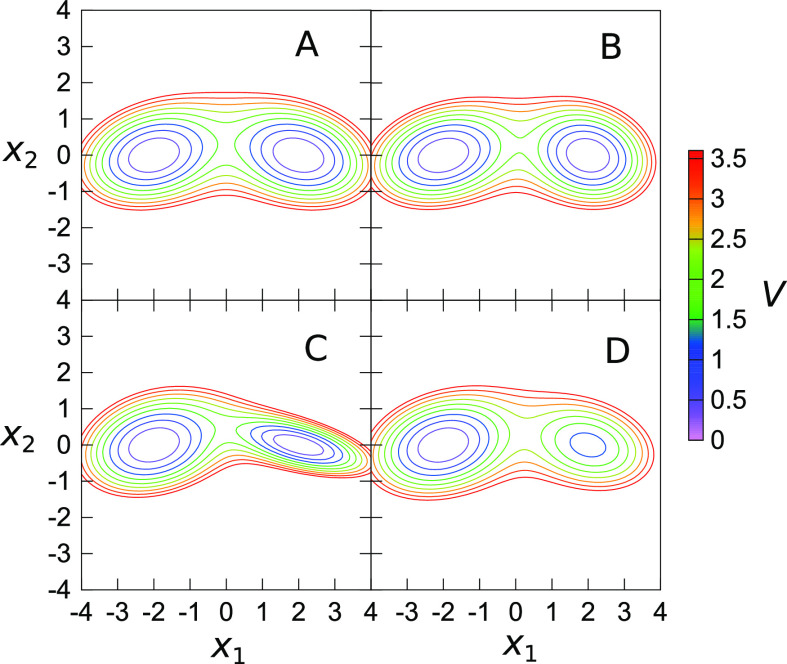
PES of the 2D model given by eq 10. Top left panel corresponds to the symmetric potential, top right to ω_1_^(R)^ > ω_1_^(L)^, bottom left to ω_2_^(R)^ > ω_2_^(L)^, and bottom right to *d* > 0, with other parameters set equal to the symmetric case.

**Figure 4:**
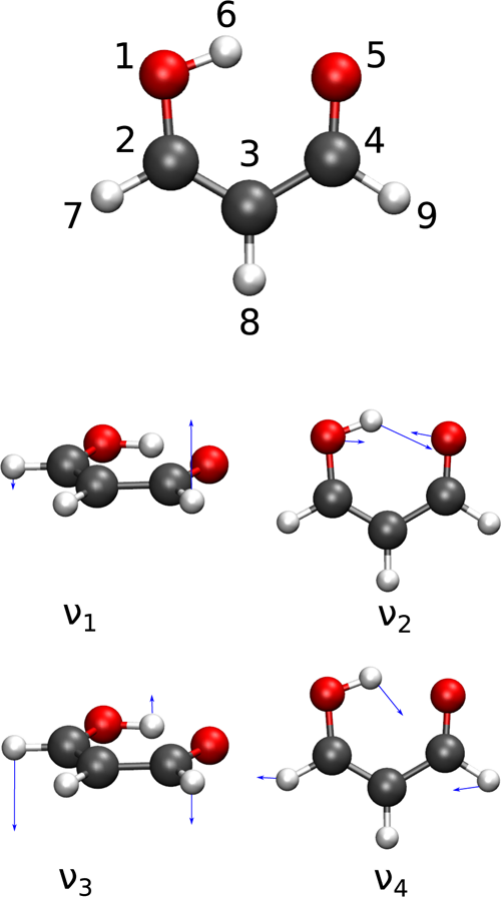
Annotated equilibrium geometry of malonaldehyde and schematic representation of four lowest-frequency normal modes.

The asymmetry in molecules can also be found in some tautomers. In this case, the potential energy surface (PES) does not possess a symmetry relating the wells and their shapes, and the minimum energies are different. A possible candidate belonging to this class is 2-hydroxy-1-naphthaldehyde, shown in Figure 1. The hydroxyl proton forms a hydrogen bond with the oxygen atom of the carbonyl group and can tunnel to it to form a tautomer, which is a local minimum.

Thiomalonaldeyde has two nearly degenerate minima in the form of enol and enethiol tautomers, shown in Figure 2. Enethiol is about 70 cm^–1^ more stable,^15^ with the barrier height to interconversion slightly lower than in the malonaldehyde. This implies that the TS is similar in magnitude to the energy asymmetry of the wells and the states in different wells that lie below the barrier are expected to interact. Interestingly, it has been suggested^15^ that the replacement of hydrogen, shared by the hydrogen bonds OH–S and SH–O, by deuterium reverses the stability order of tautomers due to the zero-point energy effect.

The asymmetry can also be caused by the environment. Molecules in rare gas matrices can have energy asymmetry between the wells comparable to their TS in isolation. Delocalization of the tunneling hydrogen was observed^16^ in 9-hydroxyphenalone embedded in a neon matrix. Molecules in crystals in the vicinity of a suitable guest molecule can also have comparable energies of the splitting and energy asymmetry of the wells.^17^

Quantum tunneling has also been observed in macroscopic systems. Tunneling of Bose–Einstein condensates,^18^ electron spin tunneling in the nanomagnetic molecules,^19^ or tunneling of the magnetic flux in superconducting circuits based on Josephson junctions^20^ are some recent examples. In a collective macroscopic variable, these processes can be described by a double well with externally controllable parameters that can induce asymmetry between the wells.

Calculation of the TS in moderately large molecules is prohibitively costly. Exact variational methods for determining the bound states of molecules scale exponentially with the basis set size, while large basis sets are often required.^21^ Basis functions need to span over two or more wells sufficiently densely to obtain enough resolution to extract the splittings from the difference of the energies in their spectrum. The asymmetry of the wells also suggests that the symmetry cannot be used to reduce the size of the problem. Full-dimensional studies of malonaldehyde using multiconfigurational time-dependent Hartree^22,23^ (MCTDH) and variational calculations on the HF dimer^21^ or H_2_O dimer^24^ represent the state-of-the-art calculations of the vibrational levels using formally exact methods.

A direct calculation of the TS in larger systems can be performed using a recently developed path integral molecular dynamics method^25^ based on the potential sampling around the minimum action paths (MAPs) connecting different wells. The multiwell splitting patterns of the water trimer and hexamer^26^ were obtained in this way using a matrix model of Hamiltonian in the basis of local vibrational states. The tunneling matrix (TM) elements are extracted from the zero-temperature limit of the partition function, which means that the method only works for the vibrational ground state in symmetric well systems.

Alternatively, the TM elements can be estimated using semiclassical methods. From that class, the instanton method, which comes in several forms,^27−30^ has some particularly appealing features. It can be applied in Cartesian coordinates^29,31^ to any molecule without modification. Numerically, it relies on the optimization of the MAP that connects the symmetry-related minima^32^ and requires the potential and Hessians of the potential along the MAP to evaluate the splittings. It thus relies on a modest number of potential and gradient evaluations^33^ in comparison with the exact quantum mechanical (QM) methods. This allows one to perform calculations in full dimensionality or in combination with on-the-fly evaluation of the electronic potential. Additionally, its accuracy is higher for large barriers and small splittings. Precisely in this regime, the exact variational methods become inefficient and resource-intensive.

The first derivation of the multidimensional instanton theory was accomplished by means of Jacobi fields integration (JFI).^30^ The JFI method has been used to determine TSs for a range of symmetric double-well systems, such as malonaldehyde,^30,32,33^ the vinyl radical,^34^ and the formic acid dimer.^35^ The instanton method was later rederived in the ring polymer form (RPI),^29^ which could treat asymmetric potentials along MAPs and multiple wells. The RPI was used to calculate and interpret experimental ground-state splitting patterns of water clusters in terms of their rearrangement dynamics^8^ for the dimer,^29,36,37^ trimer,^29,38^ hexamer,^39^ and octamer.^40^ We recently generalized the JFI method^31^ to treat the multiwell systems and used it to explain the ground-state splitting pattern of 320 states in the water pentamer in terms of five dominant rearrangement pathways.^41^ The extension of the method to low-lying vibrational states^42^ is based on the work of Mil’nikov and Nakamura^43^ and forms the groundwork of calculating the TM elements between local vibrational states of different wells in the present study below.

Weakly biased double-well systems have been considered in previous studies by several authors. Analytical results in one dimension have been obtained using the semiclassical Wentzel–Kramers–Brillouin (WKB) and instanton methods. Garg has demonstrated^44^ that the instanton method and the WKB method with the Herring formula^45^ give equivalent results for the TS in symmetric systems. Cesi et al.^46^ considered a one-dimensional (1D) double-well with the shape asymmetry and no energy asymmetry using instantons and obtained an expression for the ground-state TS. An approximate solution for a 1D double well with a weak bias was also obtained by Mugnai and Ranfagni,^47^ using instantons based on the MAP that does not fully connect the minima of the two wells. Leggett et al. obtained a solution^48^ by adding a parabolic correction potential to remove the asymmetry between the wells, the contribution of which was then subsequently subtracted from the action integral. Dekker^49^ derived the ground-state TS from the quantization condition via asymptotic matching of the semiclassical wavefunction in the barrier to the parabolic cylinder wavefunctions of harmonic oscillators in the two wells. Song^50,51^ extended Dekker’s method^49^ (as Halataie and Leggett^52^ have performed independently) to obtain the TS in vibrationally excited states of asymmetric 1D potentials with an arbitrarily large shape and energy asymmetry. Song also showed^51^ that the instanton wavefunctions with the Herring formula in a 2 × 2 matrix model give equivalent results to those obtained using Dekker’s method.^49^

In multidimensional systems, tunneling can be assisted or supressed by the excitation of transversal vibrational modes.^43,53^ In the presence of asymmetry, the excited states of one well can be in a resonance with the states of another well with a different set of local quantum numbers, which results in a delocalization of the wavefunction across these wells.^51^ Benderskii et al. devised a multidimensional perturbative instanton method^54^ in which they treat the asymmetry of the potential in an analytic two-dimensional (2D) model as a correction of first order in ℏ, the same as that for energy. In this way, the MAP remains symmetric, and the asymmetry is moved to the transport equation along with energy. They also show that the equivalent expressions for the TS are obtained using the instanton quantization condition of Dekker^49^ and using the instanton or WKB wavefunctions with the Herring formula^45^ in one dimension. The method was applied to calculate TSs in excited vibrational states of malonaldehyde^55^ with the asymmetric isotopic substitutions using a fit of model potential parameters to quantum-chemical data. The extensions of the RPI and JFI methods to the ground-states of the asymmetric systems with a weak bias have recently been derived and applied to PD malonaldehyde^9^ and the water trimer,^14^ respectively.

The object of this paper is to propose a method for calculating the vibrational tunneling spectrum of multiwell systems of mid-sized molecules that are outside the reach of the exact quantum methods. For this purpose, we extend the usual 2 × 2 matrix model to the ∑_*m*_*N*_*m*_ × ∑_*m*_*N*_*m*_ model, which represents the molecular Hamiltonian in the basis of all *N*_*m*_ local vibrational states of each well *m*. We rederive a generalized Herring formula^45,54^ in order to calculate the off-diagonal TM elements that represent the interaction of local vibrational states of different wells. The semiclassical wavefunctions at the dividing plane, in the barrier that separates the wells, are obtained using the recently generalized JFI method.^31,43^ The JFI wavefunctions are thus used to calculate the couplings between states that have different energies and normal-mode excitations for the first time. The diagonal energies of the local vibrational states can be calculated using any accurate quantum method with a basis set that spans only one well. Vibrational configuration interaction^56−58^ (VCI) is used in this work. The effect of rotations is neglected.

The method, presented in Section 2, allows one to study the vibrational structure in asymmetric systems with multiple wells, separated by large potential barriers, in an approximate manner. The accuracy of the method is tested on a 2D double-well model in Section 3.1. In Section 3.2, it is applied to the (symmetric) malonaldehyde molecule, which tests the accuracy of the matrix model using a combination of VCI and JFI matrix elements on a realistic PES, in vibrationally excited states, against the exact MCTDH calculations. The vibrational tunneling spectrum of PD malonaldehyde is calculated in Section 3.3, which features the mixing of inequivalent well states due to tunneling. The paper concludes in Section 4.

## 2 Tunneling Matrix

Without the loss of generality, we start by considering a system with two minima separated by a large potential barrier. The minima, denoted as “left” (L) and “right” (R), are not necessarily symmetric either in shape or energy. For low-energy spectra, the vibrational Hamiltonian can be represented in the basis of states that are localized in the wells, {ϕ_*i*_^(L)^,ϕ_*j*_^(R)^}, as
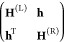
1Square blocks **H**^(L/R)^ are formed using basis functions of the same minimum and are not necessarily of equal size. Their off-diagonal elements describe the interaction between different basis functions localized in the same minimum and can be made small by a suitable choice of the basis. In the instanton theory of TSs, the usual presumption is that the local vibrational wavefunctions are harmonic oscillator states. In that case, the off-diagonal terms describe anharmonic contributions that originate from the difference between the actual potential and the harmonic potential.

In our approach here, we replace the harmonic surface of each well by an *n*-mode representation^59,60^ of the well potential and calculate local eigenfunctions and eigenvalues using the vibrational self-consistent field (VSCF) and VCI methods.^56−58^ The technical details of the calculations are described in Section 2 of the Supporting Information. Using the more accurate local wavefunctions as a basis reduces the magnitude of the off-diagonal matrix elements in **H**^(L/R)^, which we then neglect. The matrices **H**^(L/R)^ become diagonal, and the diagonal matrix elements are referred to as the local vibrational energies of the left/right (L/R) well. For symmetric wells, the local energies are doubly degenerate.

The block **h** in matrix (1) contains the TM elements that describe the interaction of local wavefunctions of the left and right minima. The exact quantal calculation of these elements requires a large basis set that can accurately represent the form of the wavefunction inside the barrier. Instead, we obtain them by means of the Herring formula^45^ in combination with the semiclassical wavefunctions from the instanton theory.^42,43^ Since the only effect of matrix (1) is to mix local wavefunctions of different minima via tunneling, we refer to it as the TM.^29^

We now derive the Herring formula without the usual assumptions of the two-state model and the L/R symmetry. Rather, we consider the Schrödinger equation with Hamiltonian (or tunneling) matrix (1), from which it follows that
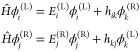
2where *E*_*i*_^(L/R)^ are the local vibrational energies and the summation over repeated index *k* is assumed. We proceed in mass-scaled Cartesian coordinates and define a dividing plane inside the barrier via the implicit equation *f*_D_(**x**) = 0, which separates the left minimum from the right minimum. Equation 2 are multiplied by ϕ_*j*_^(R)^ and ϕ_*i*_^(L)^, respectively, subtracted, and integrated over the left part of the domain (i.e., over the space on the “left” side of the dividing plane). The local wavefunctions ϕ_*i*_^(L/R)^, either harmonic or VCI, have been obtained as eigenfunctions of a Hermitian matrix and are therefore taken to be orthonormal. For a sufficiently high barrier, the wavefunctions ϕ_*i*_^(L/R)^ can be considered small in the R/L domain, respectively. We thus neglect the integrals involving the like products ϕ_*i*_^(R)^ϕ_*i*_^(R)^ in the L volume and extend the integrals involving ϕ_*i*_^(L)^ϕ_*j*_^(L)^ over the entire domain to produce δ_*ij*_. The integrals involving the mixed products ϕ_*i*_^(L)^ϕ_*j*_^(R)^ have also been neglected. The error introduced by the neglect of these terms outside the resonance, that is, for *E*_*i*_^(L)^ ≠ *E*_*j*_^(R)^, is analyzed in the Appendix. The TM element is then expressed as
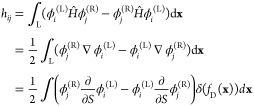
3where we exploited the use of mass-scaled Cartesian coordinates and, in the last step, used the divergence theorem to turn the spatial integration into the integral over the dividing plane. *S* in eq 3 denotes the coordinate that describes an orthogonal shift from the dividing plane.

Local wavefunctions, which we designed to calculate the local vibrational energies on the diagonal of matrix (1), are constructed using the VSCF/VCI on an approximate PES (see Section 2 in the Supporting Information), and their accuracy drops inside the barrier that separates the wells. In order to evaluate the surface integral in the Herring formula, eq 3, inside the barrier, we employ the JFI wavefunctions instead, which we recently derived in ref (42). These are based on the WKB method in which the energy is treated as a term of order ℏ^1^ and is moved to the transport equation, leaving the Hamilton–Jacobi equation energy independent. It was shown that this approach gives equivalent results to the standard WKB method in one dimension.^44^ Moreover, the ground-state TS obtained from the Herring formula using the ground-state JFI wavefunctions^42^ is identical to the standard instanton result derived from the steepest descent approximation of the partition function in the path integral formulation.^31^

The characteristic of the Hamilton–Jacobi equation that connects the minimum of a well to a point in the configuration space obeys the equation^42^
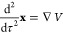
4and represents a classical trajectory **x**(τ) on the inverted PES, parameterized by the “imaginary” time τ. In order to represent the quantities in the neighborhood of the characteristic, *N* local coordinates (*S*, Δ**x**) are defined,^42,43^ where *S* is the mass-scaled arc length distance from the minimum along the characteristic and Δ**x** is the orthogonal shift from the nearest point on the characteristic. The classical momentum is defined as
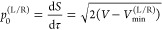
5and *S* can be used, instead of τ, to reparameterize the characteristic. Local wavefunctions in the harmonic vicinity of the characteristic are obtained by integrating the Hamilton–Jacobi and transport equations on the characteristic^42^ as
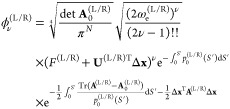
6For vibrationally excited states, label ν in eq 6 represents the number of quanta in the excited vibrational mode of frequency ω_e_. Matrices **A**^(L/R)^ are Gaussian widths of the wavefunction in the directions orthogonal to the characteristic and are obtained from
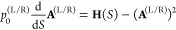
7**H**(*S*) in eq 7 is the Hessian of the potential at *S*, which is used to approximate the potential up to quadratic terms in the neighborhood of the characteristic. The initial condition for eq 7 at the minimum is **A**_0_^(L/R)^ = (**H**_0_^(L/R)^)^1/2^, where **H**_0_^(L/R)^ is the Hessian at the L/R minimum. For vibrationally excited states, the prefactor in the parentheses in eq 6 contains terms *F*(*S*) and **U**(*S*), which are defined via equations
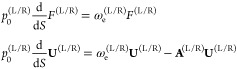
8*F*^(L/R)^ terms account for the change in the amplitude of the excited-state wavefunction along the characteristic, while the **U**^(L/R)^ term describes the nodal plane. The initial condition for *F*^(L/R)^ is found by matching the instanton wavefunction to that of the harmonic oscillator at a small distance S = ε from the minimum as *F*^(L/R)^ (ε) = **U**_0_^(L/R),T^(**x**(ε) – **x**_min_^(L/R)^). The **U**_0_^(L/R)^ is the excited-state normal mode, that is, the eigenvector of **H**_0_ having frequency ω_e_, and serves as the initial condition for **U** in eq 8.

The local instanton wavefunctions, eq 6, for the left and right minima are next inserted into the Herring formula given in eq 3 without the previous assumption^42^ that ϕ^(L)^ and ϕ^(R)^, each, refer to the excitation of the same normal mode and the same number of quanta ν. For this purpose, a connection point **x**(*S*_cp_) is chosen on the dividing surface *f*_D_(**x**) inside the barrier, and characteristics are determined, which connect it to the minima on both sides of the dividing surface. The shape of the characteristic between two points in the configuration space is determined by minimizing the Jacobi action.^32^ The surface integral in eq 3 can then be computed analytically.^42^

This approach yields the best results if the connection point is chosen so that both wavefunctions are near their maxima in the dividing plane at the connection point. This can be obtained by the minimization of the sum of action integrals . For minima of the same energy, this procedure yields the MAP that connects the minima and any point on that path is a suitable candidate for the connection point. The dividing surface can then be chosen as the plane orthogonal to the MAP at the connection point. If the minima do not have the same energies but differ by the amount *d*, this procedure is equivalent to determining the MAP on the modified PES *Ṽ*(**x**) = *V*(**x**) – Θ(*S* – *S*_cp_)*d*, where *S*_cp_ is the position of the connection point on the characteristic and Θ is the Heaviside step function. In this case, the position of the connection point has to be given a priori, and the resulting path will depend on its position. The safest choice is to pick the connection point in the middle of the MAP, which is expected to be near the maximum of the potential energy barrier. For minima at different energies, the resulting MAP is going to have a tangent dicontinuity at the connection point as *p*_0_^(L)^ ≠ *p*_0_^(R)^ at *S*_cp_. The tangent direction at the connection point is then defined as the average tangent of its L and R limits at *S*_cp_. Again, the dividing plane is taken to be orthogonal to the MAP, and the surface integral in eq 3 is solved analytically. The TM element then becomes
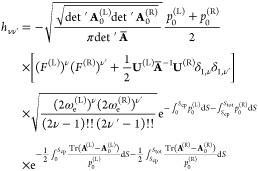
9with all quantities in the brackets evaluated at *S* = *S*_cp_. In eq 9, *S*_tot_ is the total length of the MAP, , and the symbol ⊥ means that the tangent direction to the MAP was explicitly projected out. The det′ in eq 9 denotes the product of all non-zero eigenvalues. Matrices **A**_0_ have zero eigenvalues associated with the overal translations and rotations, while **A̅** has an additional zero eigenvalue associated with the tangent to the MAP. For energy-equivalent minima, the tangent vector is an eigenvector of **A̅** with zero eigenvalue, and the explicit projection to the orthogonal space is not needed. The TM element in eq 9 is valid for ν, ν′ = 0–1. For ν > 1 and multiple excitations in different modes, the TM element can still be evaluated using the Herring formula and wavefunctions of form (6) using analytical integrals, but we have only implemented it numerically, without trying to write down the explicit form. We also remark here that the wavefunction in eq 6 for the multiply excited normal modes, ν > 1, does not correspond to the harmonic oscillator wavefunction near the minimum as the prefactor in eq 6 is not a Hermite polynomial. We further note that the TM element, (9), is not invariant with respect to the position of the connection point *S*_cp_ unless the two local states are in resonance, as shown in the Appendix.

## 3 Numerical Tests

Numerical tests were carried out on a model 2D PES and on the malonaldehyde molecule with some atoms substituted with heavier isotopes. MAPs that connect the minima were determined using the string method.^32,33^ The criterion for convergence was chosen to be the largest component of the gradient of Jacobi action perpendicular to the path and was set to be 10^–6^ au. The number of beads used to discretize the string was 301 for model potential, which is much larger than necessary for convergence but was used to ensure that all results obtained using different parameters of the potential are sufficiently converged. For the potential with minima at different energies, the dividing plane was set to pass through the central bead and perpendicular to the MAP. In the case of malonaldehyde, the number of beads was 201, and the minima were oriented toward the first neighboring bead in each step of the optimization to minimize the root-mean-square distance between their geometries.^32^ After optimization, Hessians of the potential were determined at each bead on the MAP. Translations and rotations were explicitly projected out from Hessians.^31^ Geometries along the path in mass-scaled Cartesian coordinates, potential, and Hessian matrix elements were parameterized by the arc length *S* along the MAP and interpolated using natural cubic splines. Matrices **A**^(L/R)^ in eq 7 were propagated using the previously described approach,^42^ with the initial “jump” at ε = 0.1 au for model potential and ε = 0.25 au for malonaldehyde. The fourth order Runge–Kutta method was used for integration of eq 7. Matrices **A**^(L/R)^ (*S*) were saved at each bead, and their matrix elements were interpolated using natural cubic splines, as implemented for the Hessians above. The interpolant was then used to propagate *F*^(L/R)^ and **U**^(L/R)^ in eq 8 from minima up to the dividing plane.

The particular implementation of the VSCF/VCI method that is employed in our calculation here is described in Section 2 of the Supporting Information. We determined the 1-mode and 2-mode terms of the PES and neglected the terms beyond. In each normal mode, the potential was evaluated at Gauss–Hermite discrete variable representation (DVR) points, which correspond to the zeroes of Hermite polynomials. We used eight DVR points for the 2D model potential and 11 DVR points for malonaldehyde. This approach utilizes the natural lengthscales of the harmonic oscillators in each normal mode, which gives a balanced description of the potential at different minima. The 1-mode terms were then fitted to the eighth-order polynomials using linear regression. For 2-mode terms, the potential was computed on a rectangular grid of DVR points determined above, and a fit was performed analogously. For each 1-mode potential, a quick QM calculation was performed using sine DVR basis with 100 basis functions. The difference in the lowest two energies from that calculation was used as a frequency for the harmonic oscillator basis set, which was used to solve the VSCF equations. This approach provides a better basis for determining the 1-mode potentials that quickly deviate from the harmonic curve, reducing the number of basis functions needed to describe the 1-mode functions in the VSCF. The basis sets of 7 and 16 harmonic oscillator functions in each normal mode in the 2D model potential and malonaldehyde, respectively, were needed to converge the energies (to less than 1 cm^−1^ for the VSCF ground state). The existence of a plateau over which the energies of interest do not change appreciably with the basis set size, in comparison with the size of the TM elements, indicates that the approximate separability is possible, which is a necessary condition for the applicability of the proposed combined approach. A larger basis should not be used as functions corresponding to larger energies start to penetrate into unphysical parts of the fitted potential, which can cause the appearance of intruder states and worse energies. After VSCF calculation, the computed 1-mode functions were used for VCISD calculation, where the highest excitation in each mode was limited to six in both the 2D model system and malonaldehyde.

### 3.1 2D Model Potential

The 2D model potential with two minima, which we use in our test calculations below, is defined by the following equations
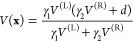

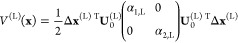

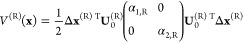

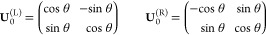

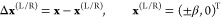

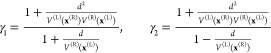
10where **x** are not mass-scaled. Minima are located at **x**^(L/R)^. Coefficients γ_1_ and γ_2_ are chosen so that in the vicinity of the left minimum, the potential is approximately harmonic and equals *V* ≈ *V*^(L)^, while in the vicinity of the right minimum, the potential is approximately harmonic and shifted in energy by *d*, that is, *V* ≈ *V*^(R)^ + *d*. α_1,L/R_ and α_2,L/R_ are eigenvalues of the Hessian, while **U**_0_^(L/R)^ are normal modes. Parameter θ denotes the angle of inclination of the normal mode to the *x* axis. The mass of the system was taken to be *m* = 3.5 in both dimensions so that the harmonic frequencies are given by .

The above form of the potential can be used to independently vary harmonic frequencies ω_1/2_^(R)^, by changing parameters α_1/2,R_, or the shift *d* without affecting the other parameters of either the left minimum or the right minimum. In this paper, the parameters of the left minimum were α_1,L_ = 1.6 and α_2,L_ = 4.0. The parameters of the right minimum were the same as the parameters of the left, for the symmetric case with *d* = 0. To obtain the asymmetric potentials below, one of the three parameters was varied, with α_1,R_ going from 1.6 to 36, parameter α_2,R_ going from 4 to 49 and *d* going from 0 to 1.1. Positions of the minima were set with β = 2.0 and angle θ = π/12. This angle corresponds to approximately equal contributions of *F*^(L/R)^ and **U**^(L/R)^ in the TM elements.^42^Figure 3 shows the model potential for a selection of parameters α_1,R_, α_2,R_, and *d*.

**Figure 5:**
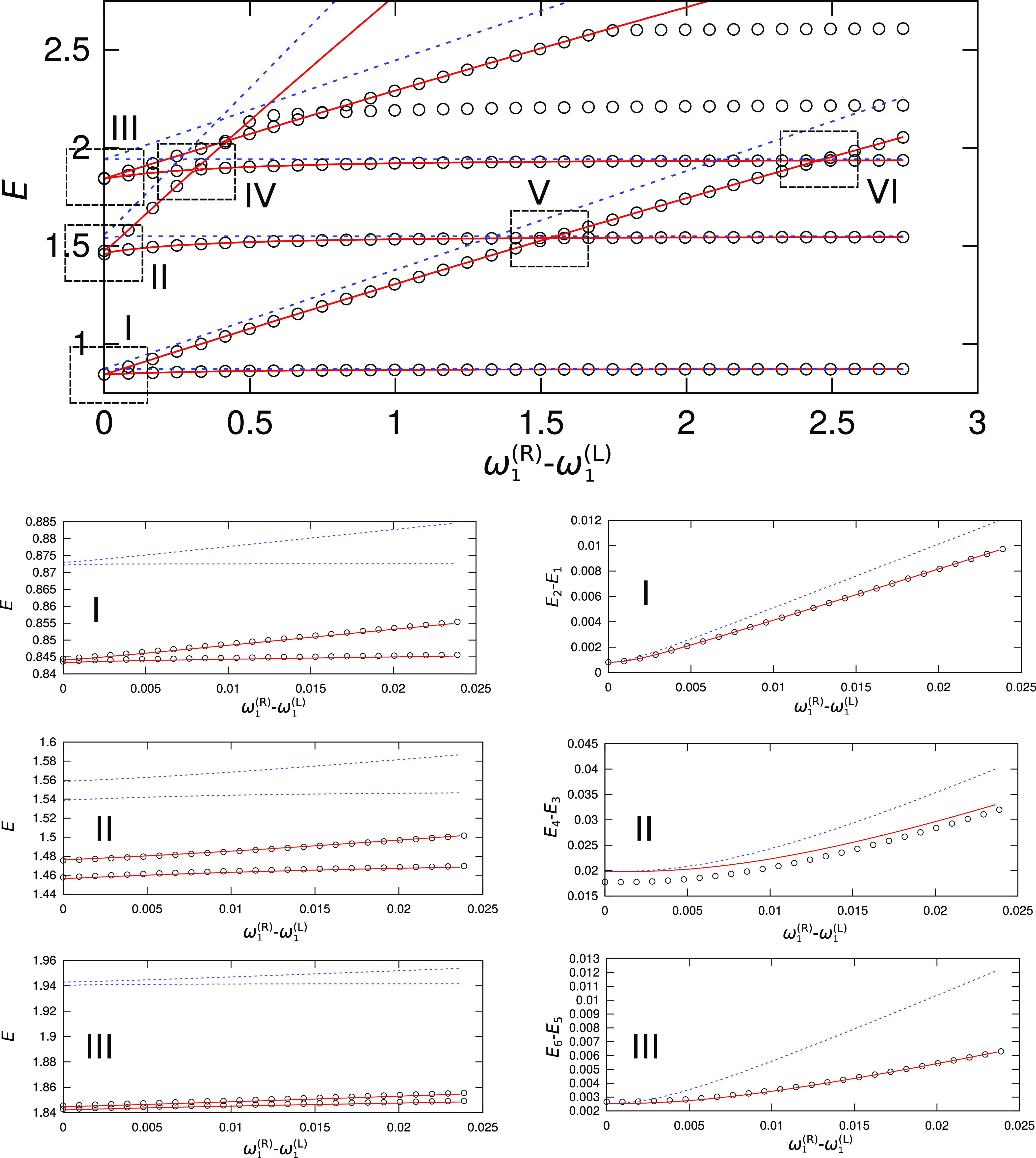
Dependence of vibrational energies of the lowest six states in the double-well potential given by eq 10 on ω_1_^(R)^. Circles represent QM values, blue lines are obtained using the instanton method with harmonic energies, and red lines are obtained using a combined VCI/instanton approach. Frames I–III in the top panel are shown magnified in the left column panels below, and the dependence of the associated TSs on ω_1_^(R)^ is shown in the right column panels below.

Frequency ω_1_ is the lower frequency, and the MAP enters the minima along the corresponding normal mode. Consequently, ω_1_ does not contribute toward the zero-point energy in the plane orthogonal to the MAP. The effective barrier for the tunneling motion from the ground state in the left minimum, corrected by the zero-point motion contribution, can be defined as
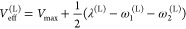
11*V*_max_ in eq 11 is the maximum of the potential *V*(*S*_max_) along the MAP. λ^(L)^ is the non-zero eigenvalue of the **A**_⊥_ = **PAP** matrix, where **P** projects out the tangent direction to the MAP at *S* = *S*_max_. The effective barrier can be defined for other states similarly. Figure 5 (in the second column panels) shows that for the symmetric case, ω_1_^(R)^ = ω_1_^(L)^, the JFI theory provides accurate TSs in the ground state, and in the second excited state, which corresponds to the excitation of the transversal frequency ω_2_. In the first excited state, the JFI theory slightly overestimates the TS. In that state, the effective barrier is much smaller and equals *V*_eff_ = 0.545, in contrast with the barriers of 1.221 and 0.976 for the ground and second excited states, respectively. This overestimation is a known property of the instanton method.^42^In the symmetric case, the only contribution to the splitting comes from the off-diagonal matrix elements so that the harmonic and VCI energies yield the same results. However, it can be observed (from the first column panels in Figure 5) that the harmonic vibrational energies overestimate the exact QM energies by 3–5%.

As the frequency ω_1_^(R)^ is increased and the difference in the local L/R energies begins to contribute to the overall splitting, the TSs computed using harmonic energies quickly begin to deviate from the QM values due to the neglect of anharmonicities, which no longer cancel out. When the difference in the lower frequency, ω_1_^(R)^ – ω_1_^(L)^, is only 0.02, which corresponds to the asymmetry (Δω_1_/ω_1_^(L)^) of 3%, the error in the TS of the ground state is 24%, whereas it is 90% for the transversal mode (ω_2_) excitation. A larger error in the excited state reflects the fact that the local excited-state wavefunction penetrates deeper into the barrier, where anharmonicity is larger. However, the VCI energies correctly account for the anharmonicity and provide an excellent agreement, as can be observed in Figure 5, both in the absolute energies (first column panels in Figure 5) and in the TSs (second column panels).

**Figure 6:**
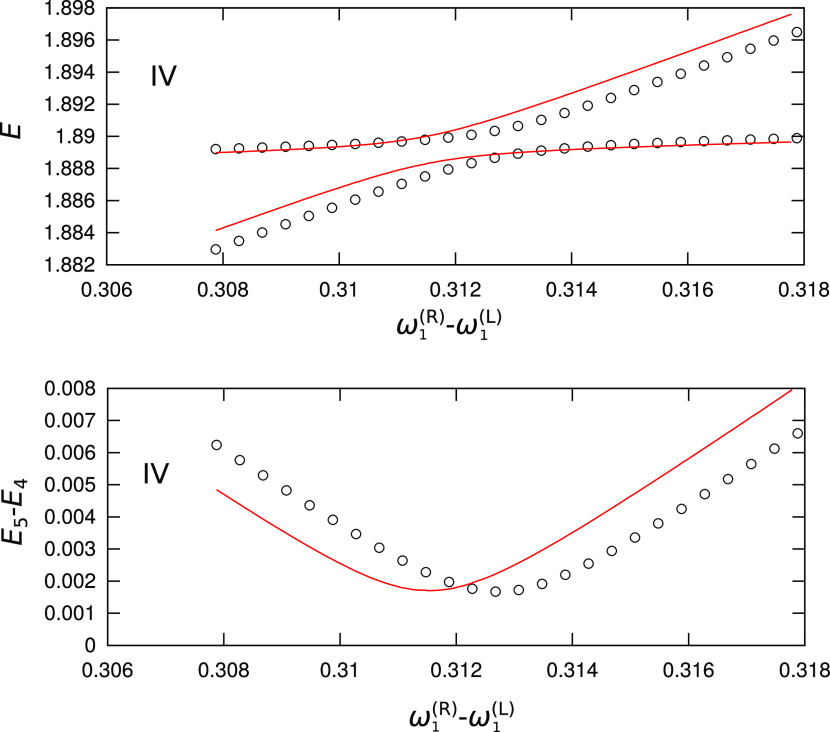
Dependence of vibrational energies and TSs in the 2D model potential given by eq 10 on the frequency ω_1_^(R)^ in the region of the avoided crossing between the first (ω_1_^(R)^-) excited state in the right minimum and the second (ω_2_^(L)^-) excited state in the left minimum, shown in frame IV in the top panel of Figure 5. Circles represent QM values, while red lines represent values obtained using a combined VCI/instanton approach.

With a further increase in the frequency ω_1_^(R)^, different local vibrational states of the left and right minima enter into resonance, and vibrational energies exhibit avoided crossings, as shown in frames IV–VI of the top panel in Figure 5. Harmonic energies do not provide accurate positions of these avoided crossings, as seen in Figure 5, due to errors in the local energies. In the case of the avoided crossing between the higher-frequency ω_2_^(L)^-excited state of the left minimum and the ω_1_^(R)^-excited state of the right minimum, as shown in frame IV of the top panel in Figure 5, the error in the position of the avoided crossing (in ω_1_^(R)^ – ω_1_^(L)^) using harmonic energies is 16% (and falls outside frame IV in Figure 5). VCI energies, as shown magnified in Figure 6 together with the exact QM energies, provide a significantly more accurate position with the error of only 0.4%. The small discrepancy can be attributed to the fact that as frequency ω_1_^(R)^ is increased, the local wavefunction in the right minimum penetrates deeper into the barrier. In this region, the approximate *n*-mode representation of the potential used in the VCI calculations begins to deviate from the actual potential, which introduces an error in the local energies.

The TS in the avoided crossing is reproduced with great accuracy, shown as the minima in the lower panel in Figure 6, with the error of 5%. Errors in the positions of other avoided crossings (IV–VI in Figure 5), namely, between the ground state of the right minimum and the ω_1_^(L)^- and ω_2_^(L)^-excited states in the left minimum (frames V and VI in Figure 5, respectively) become larger even using VCI energies. The local wavefunction of the right minimum has a larger energy and penetrates deeper into the region where the *n*-mode representation of the potential becomes unreliable. Nevertheless, the TSs in the avoided crossings are again reproduced accurately, which indicates that the JFI method can indeed give reliable TM elements between different vibrational states of L/R minima and, in combination with the VCI energies, is a useful tool for computing vibrational tunneling spectra. Similar results were observed with the frequency ω_2_^(R)^ varied (shown in Figures S3 and S4 in the Supporting Information).

**Figure 7:**
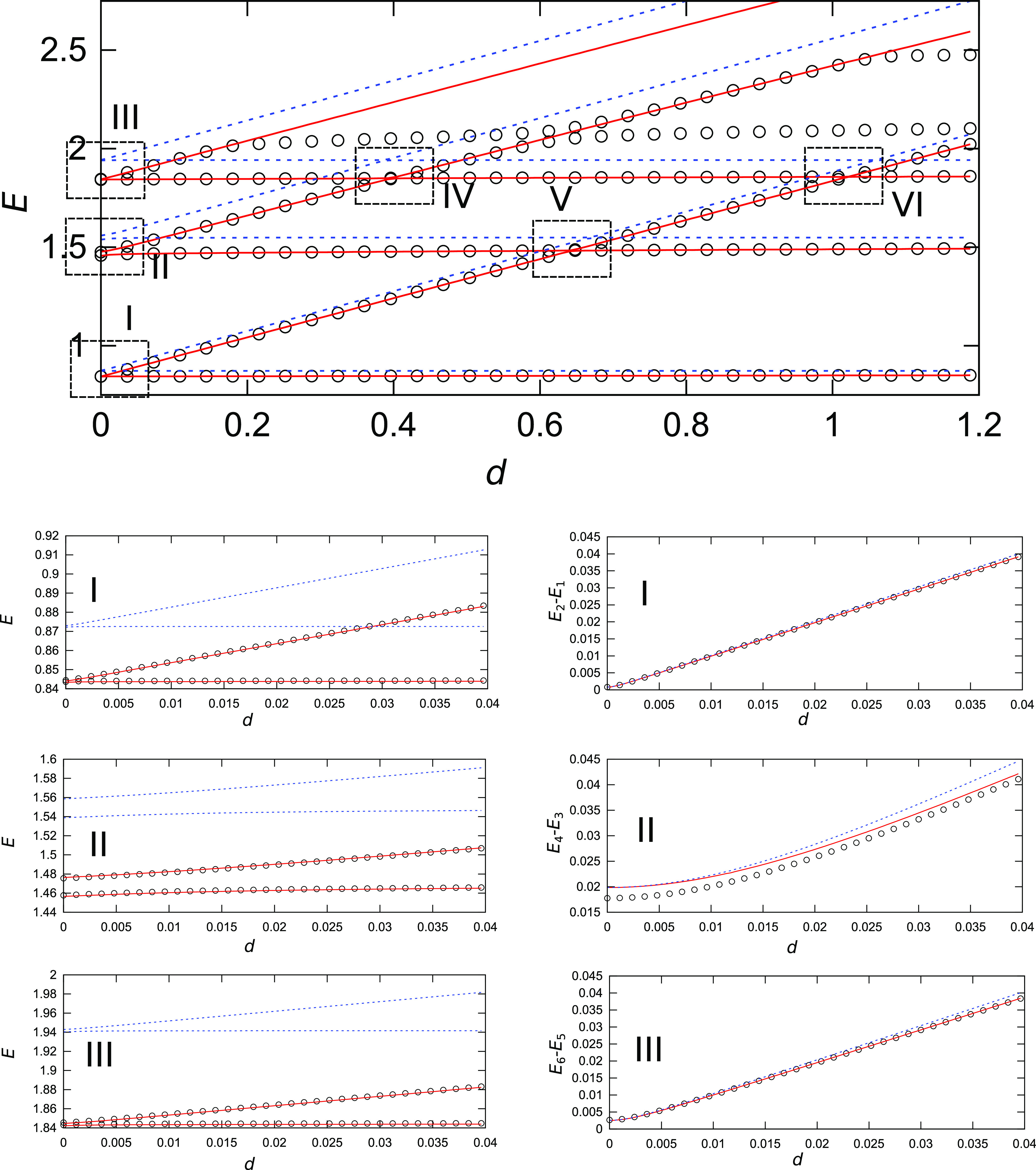
Dependence of vibrational energies of the lowest six states in the double-well potential given by eq 10 on the energy shift *d* of the right well. Circles represent QM values, blue lines are obtained using the instanton method with harmonic energies, and red lines are obtained using a combined VCI/instanton approach. Frames I–III in the top panel are shown magnified in the left column panels below, and the dependence of the associated TSs on *d* is shown in the right column panels below.

**Figure 8:**
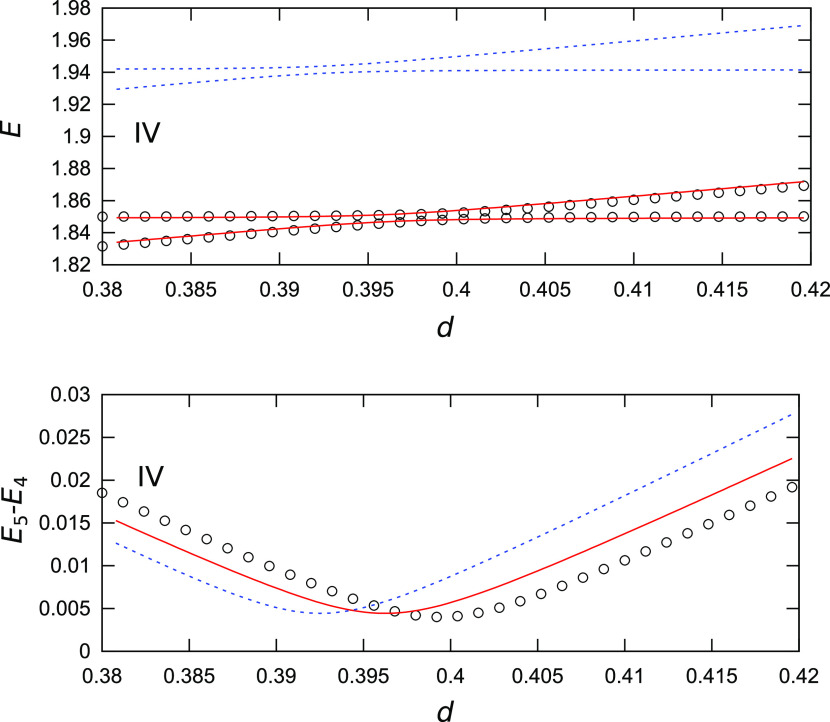
Dependence of vibrational energies and TSs in the 2D model potential given by eq 10 on the energy shift *d* of the right well in the region of the avoided crossing between the first (ω_1_^(R)^-) excited state in the right minimum and the second (ω_2_^(L)^-) excited state in the left minimum, shown in frame IV in the top panel of Figure 7. Circles represent QM values, blue lines are obtained using the instanton method with harmonic energies, and red lines represent values obtained using a combined VCI/instanton approach.

Figure 7 shows the dependence of energy levels on the variation in the depth *d* of the right minimum. Overall, the introduction of the energy asymmetry between the wells results in a similar energy level pattern to that observed above. A notable difference is that in this case, the TSs obtained using harmonic energies are much closer to the exact QM values. This is an artefact of the construction of the PES, in which the frequencies in the left and right minima are the same. As a result, the shapes of the local potentials in both minima are similar, and a large part of the error introduced by the anharmonic terms cancels out. However, in realistic applications, it is unlikely that systems with minima of different energies have the same L/R frequencies. The error in the position of the avoided crossing IV is also much smaller for the harmonic energies (≈2%), while it is further reduced using VCI energies (0.7%), as shown in Figure 8. The error in the TS in the avoided crossing is 12%, which is comparable to the error in the case of the frequency variation, as shown in Figure 6.

### 3.2 Malonaldehyde

We next employ our combined approach to study the symmetric, homoisotopic malonaldehyde on the PES developed by Wang et al.^61^ The molecule is shown labeled in the top panel in Figure 4. It has two equivalent wells with hydrogen 6 attached to either oxygen 1 or 5. We study below the effect of adding additional states in the TM. For this purpose, vibrational energies are computed either from a 2 × 2 matrix involving corresponding states in the two wells, an 8 × 8 matrix involving four local states at both sides of the barrier, or a 16 × 16 matrix model. Thereby, we again calculate the local single-well states using the VSCF/VCI, while the TM matrix elements are computed using the recently developed JFI method.^42^

Malonaldehyde has been extensively studied in the past^62^ and presents a benchmark system for the development of quantum dynamical methods. Most recent calculations on the same PES using exact quantum methods were obtained using MCTDH by Hammer and Manthe^23^ and Schröder and Meyer^22^ and show a good level of agreement with experimental results.^63^ We use the results of ref (23) for comparison as they report TSs for a number of vibrationally excited states having a large transition dipole moment and are believed to be more accurate.^22^

Local harmonic and VSCF/VCI energies, calculated in a 2-mode representation of the single-well potential, as described in Section 2 of the Supporting Information, for the lowest 8 vibrational states, that we consider below, are shown in Table 1. The ground state is labeled GS, while the excited states are labeled by the frequencies ν_*i*_ of the excited normal modes, numbered in the order of increasing frequency in the subscript and separated by a “+” sign for multiple excitations. A noticeable shift can be observed between all harmonic and VCI energies in Table 1 due to anharmonicity, but the order in energies remains unchanged. The lowest four normal modes that can get excited in the lowest eight local vibrational states and that play a role in our calculations below are depicted in Figure 4. Higher vibrational states become more densely spaced in energy and start to mix vibrational modes at minima. Our approach relies on being able to uniquely define the excited normal modes at minima for each local vibrational state considered because the instanton wavefunctions that are used to calculate the TM elements that connect these states tend to harmonic oscillator eigenstates at minima. Moreover, a higher density of states at higher energies would require the inclusion of many additional states in the TM, which are not known as precisely as for the low-lying states and would thus degrade the accuracy. We limit ourselves, therefore, to the lowest eight local states in the studies of tunneling spectra of malonaldehyde below.

**Table 1:** Harmonic and VCI Energies in Inverse Centimeters (cm^–1^) of the First Eight Local Vibrational States of Malonaldehyde Labeled by the Excited Normal Mode Frequencies^a^

state	harmonic	VCI
GS	14,950.11 (0.00)	14,682.46 (0.00)
ν_1_	15,218.68 (268.57)	15,012.65 (330.19)
ν_2_	15,245.53 (295.42)	15,042.51 (360.05)
ν_3_	15,333.29 (383.17)	15,133.95 (451.49)
ν_1_ + ν_1_	15,487.25 (537.14)	15,262.05 (579.59)
ν4	15,472.20 (522.08)	15,281.89 (599.43)
ν_2_ + ν_2_	15,540.95 (590.83)	15,318.85 (636.40)
ν_1_ + ν_2_	15,514.10 (563.99)	15,336.16 (653.70)

a Energies relative to the local ground state are given in parentheses.

The TM elements in the **h** matrix that connect the two sets of local states in the L and R wells are calculated using the JFI method and listed in Table 2. Both minima of malonaldehyde belong to the *C*_*s*_ symmetry group, and its local vibrational states can be classified according to the irreducible representation of the excited normal mode ν_*i*_ at the minimum. The *C*_*s*_ symmetry is preserved along the MAP so that the TM elements that connect normal modes of different symmetries vanish exactly, as seen in Table 2.

**Table 2:** TM Elements Connecting the First Eight Local Vibrational States of Different Minima in Malonaldehyde in Inverse Centimeters (cm^–1^)

	GS^(R)^	ν_1_^(R)^	ν_2_^(R)^	ν_3_^(R)^	(ν_1_ + ν_1_) ^(R)^	ν_4_^(R)^	(ν_2_ + ν_2_) ^(R)^	(ν_1_ + ν_2_) ^(R)^
GS^(L)^	–12.30	0.00	–21.94	0.00	–4.98	–4.62	–25.53	0.00
ν_1_^(L)^	0.00	–6.70	0.00	6.85	0.00	0.00	0.00	–11.95
ν_2_^(L)^	–21.94	0.00	–44.20	0.00	–8.87	–7.54	–55.97	0.00
ν_3_^(L)^	0.00	6.86	0.00	8.53	0.00	0.00	0.00	12.22
(ν_1_ + ν_1_) ^(L)^	–4.98	0.00	–8.88	0.00	–4.84	–1.87	–9.14	0.00
ν_4_^(L)^	–4.61	0.00	–7.53	0.00	–1.87	7.82	–8.14	0.00
(ν_2_ + ν_2_) ^(L)^	–25.53	0.00	–55.97	0.00	–9.14	–8.15	–75.86	0.00
(ν_1_ + ν_2_) ^(L)^	0.00	–11.95	0.00	12.22	0.00	0.00	0.00	–21.31

In a 2 × 2 matrix model, only the diagonal elements of the **h** matrix are used, and the degenerate vibrational states of L/R wells are split into doublets. Equivalent results are obtained using the first-order perturbation theory for degenerate states, yielding the TS of Δ_*i*_ = 2*h*_*ii*_. Energies of the GS and the first three excited states obtained in this manner already show a good agreement with the MCTDH results of ref (23), as can be seen in Table 3 (from the second column and the last column). The vibrational states are numbered in the order of increasing energy in Table 3. The wavefunction content, obtained from the eigenvectors of the TM, is listed in Table 4 and can be used to identify states in Table 3 in terms of the excited normal modes.

**Table 3:** Vibrational Energy Levels of Malonaldehyde in Inverse Centimeters (cm^–1^) Obtained Using a Combined VCI/Instanton Approach^a^

no.	*E*^(pairs)^	*E*^(4)^	*E*^(8)^	*E*^(MCTDH)^
1	14,670.15	14,668.69	14,667.08	14,671.3
2	14,694.76	14,693.54	14,692.76	14,694.8
3	14,998.31	14,999.77	14,987.74	14,941.5
4	15,005.95	15,005.60	15,005.09	15,008.2
5	15,019.35	15,018.91	15,018.54	15,014.9
6	15,086.70	15,087.92	15,077.14	15,005.4
7	15,125.42	15,125.86	15,125.14	15,108.3
8	15,142.47	15,142.82	15,142.04	15,124.6
9	15,243.00		15,249.04	
10	15,257.21		15,263.41	
11	15,266.89		15,266.12	
12	15,274.07		15,273.84	15,249.6
13	15,289.71		15,291.11	15,268.4
14	15,314.85		15,316.14	
15	15,357.47		15,358.55	
16	15,394.71		15,407.27	

a *E*^(pairs)^, *E*^(4)^, and *E*^(8)^ are energies obtained from the 2 × 2, 8 × 8, and 16 × 16 matrix models, respectively, as explained in the text. *E*^(MCTDH)^ are MCTDH energies from ref (23).

**Table 4:**
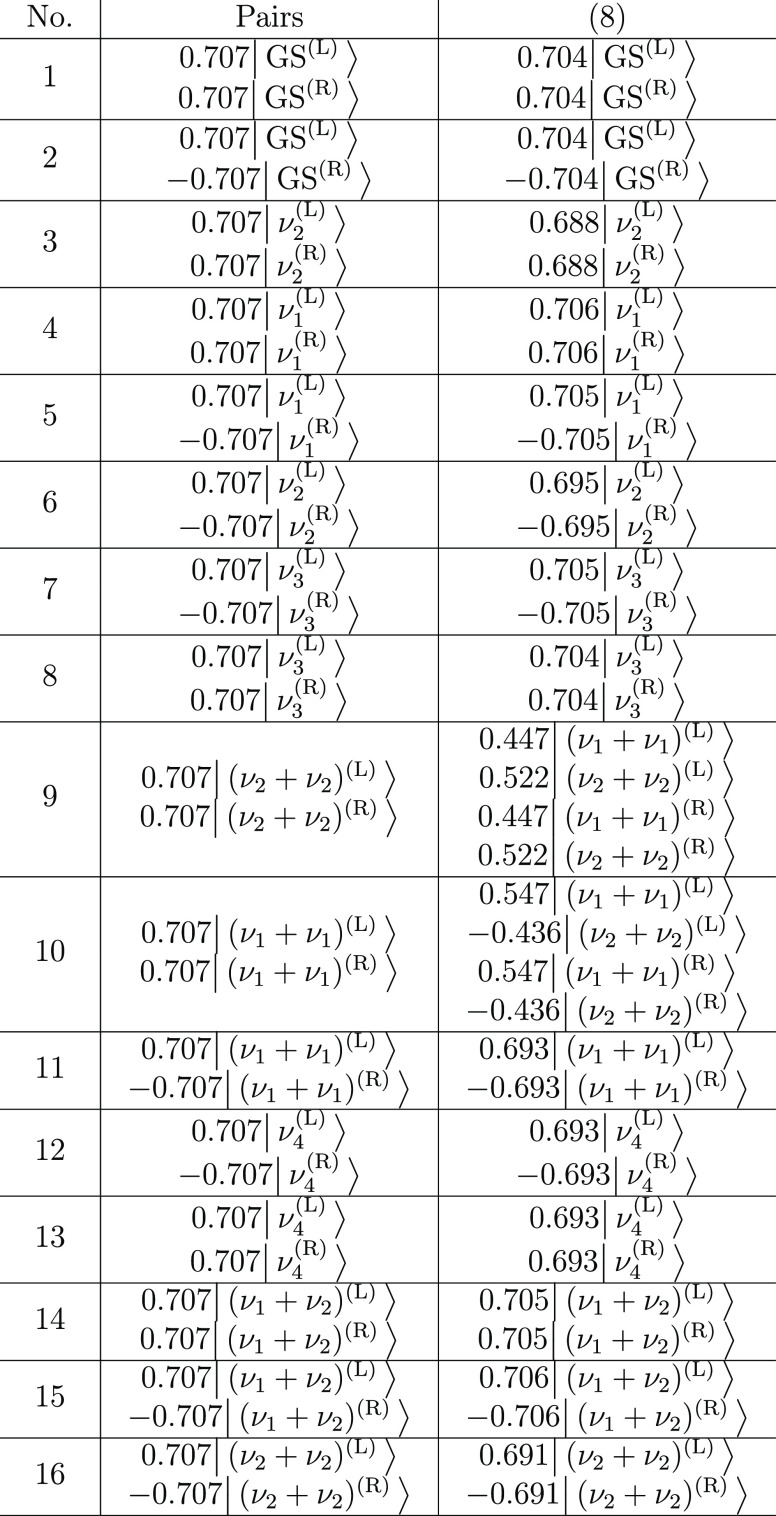
Dominant Configurations of Vibrational States of Malonaldehyde, Obtained as the Eigenvectors of the TM in the 2 × 2 (Pairs) and 16 × 16 (Eight-State) Models, as Described in the Text

The TSs for the GS and the singly excited modes ν_1–4_ in the 2 × 2 TM model are obtained as Δ(GS) = 24.60 cm^–1^, Δ(ν_1_) = 13.40 cm^–1^, Δ(ν_2_) = 88.40 cm^–1^, Δ(ν_3_) = 17.06 cm^–1^, and Δ(ν_4_) = 15.64 cm^–1^. The MCTDH results^23^ for the TSs in the same states are Δ(GS) = 23.5 cm^–1^, Δ(ν_1_) = 6.7 cm^–1^, Δ(ν_2_) = 69.9 cm^–1^, Δ(ν_3_) = 16.3 cm^–1^, and Δ(ν_4_) = 18.8 cm^–1^. Differences in TSs, apart from the ν_1_- and ν_2_-excited modes, are well within the estimated error of the MCTDH calculations, which validates the accuracy of our approach. The ν_2_ mode corresponds to the longitudinal mode as it lies parallel to the MAP at minima. The excitation of this mode effectively lowers the barrier of the tunneling motion, and the instanton theory is known to overestimate TSs in the shallow tunneling regime.^29,42^ The wavefunction also penetrates deeper into the barrier where the anharmonic effects are larger, and the VCI energies degrade as a result. Thus, the accuracy in absolute energies in Table 3 is also expected to be affected for these states. The large increase in the TS for the excitation of the longitudinal mode is, however, expected,^42^ as confirmed by our results. The TS for the ν_1_ mode is overestimated by a factor of two. This is most likely due to the anharmonicity along this normal mode, indicated by the large difference between the harmonic (268.57 cm^–1^) and VCI (330.19 cm^–1^) energies. Since the TS for the pair of states is significantly suppressed compared to the GS, the frequency and energy in its direction change substantially along the MAP. Therefore, if the anharmonicity also changes significantly, it could cause the observed discrepancy. As an aside, we also note here that the other TSs computed using MCTDH in ref (23), which do not result in the mixture of normal modes at minima, are Δ(ν_5_) = 21.1 cm^–1^, Δ(ν_7_) = 33.3 cm^–1^, Δ(ν_8_) = 14.6 cm^–1^, and Δ(ν_11_) = 19.5 cm^–1^ and are in good agreement with the values we obtained using the JFI theory as Δ(ν_5_) = 24.4 cm^–1^, Δ(ν_7_) = 39.5 cm^–1^, Δ(ν_8_) = 15.6 cm^–1^, and Δ(ν_11_) = 22.1 cm^–1^.

**Figure 9:**
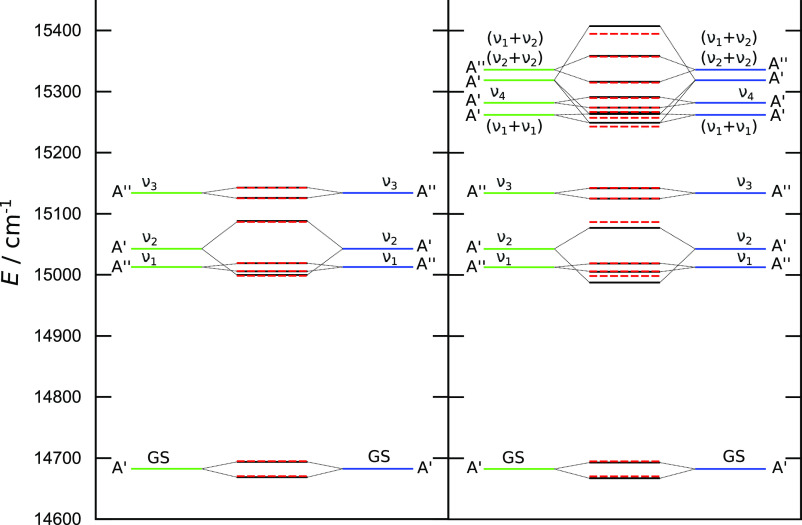
Vibrational tunneling spectrum of the lowest 8 (left panel) and 16 (right panel) states of malonaldehyde. Green and blue lines represent VCI energies of local wavefunctions in the D7 and D9 minima, respectively. Dashed red lines are obtained using a 2 × 2 TM model. Black lines in the left panel represent energies from an 8 × 8 TM model, and in the right panel, they represent energies from a 16 × 16 model. See the text for details.

We next consider the TM using four local states in each well. This takes into account interactions between the doublets considered above, whereby only the states of the same symmetry interact. If the states of the same symmetry are well-separated with respect to the size of their TM element, the shift in energy can also be computed using the second-order perturbation theory. When four local states are taken into account in the 8 × 8 TM model, slight shifts are observed in the GS and ν_2_-doublets in Figure 9 (left-side spectrum). The absolute energies change by 1.22–1.46 cm^–1^, while the perturbation theory gives the shift of 1.34 cm^–1^. However, the change in the TS is negligible.

In the 16 × 16 TM model, consisting of eight local states in each well, a strong interaction with the doubly excited (ν_2_ + ν_2_) mode causes a significant shift in the energies of the GS and the ν_2_-excited doublets as well as their splittings. The TSs change from 24.85 to 25.68 cm^–1^ and from 88.15 to 89.4 cm^–1^, which can clearly be observed in Figure 9 (right-side spectrum). A particularly strong mixing also occurs between the doubly excited (ν_2_ + ν_2_) mode and the doubly excited (ν_1_ + ν_1_) mode, for which the lower levels in the doublets are very close in energy (14.21 cm^–1^), and they interact strongly (*h* = 9.14 cm^–1^ in Table 2). The mixing results in visible changes in the dominant coefficients of TM eigenvectors in Table 4 and leads to observable energy shifts. Furthermore, the singly excited ν_4_ mode interacts and mixes with the doubly excited (ν_1_ + ν_1_) mode, which results in the change of its TS from 15.64 to 17.27 cm^–1^, which is in closer agreement with the MCTDH value of 18.8 cm^–1^. Finally, we remark that the TS of the doubly excited (ν_1_ + ν_2_) state amounts to 42.62 cm^–1^, which is in good agreement with 49.5 cm^–1^ obtained by Schröder and Meyer.^22^

The above results clearly show that the interactions of different vibrational states can have a non-negligible effect, both on the absolute values of the vibrational energies and on the values of the TSs. This effect is especially pronounced if two or more states of the same symmetry are close in energy and if the TM elements that connect them are large. This scenario is expected to play a significant role in the higher vibrationally excited states, where the density of states becomes larger and the interactions increase due to the presence of multiple excitations.

### 3.3 PD Malonaldehyde

In the previous subsection, we have learned what accuracy one might expect in the calculation of the tunneling spectra of malonaldehyde through comparison with the exact QM results. We now consider the PD malonaldehyde, where hydrogen in position 7/9 is substituted by deuterium (see Figure 4) and the system is no longer symmetric. Since deuterium is not placed in equivalent positions in the two minima, their local vibrational frequencies and energies are no longer equal, even though the PES remains unchanged. The particular choice of deuteration was chosen for our study because the mixing angle in its GS was determined experimentally by Baughcum et al.^10^ and the TS by Jahr et al.^9^ using the RPI method. Furthermore, the size of the relative energy shifts between the left and right minima is comparable to the size of the TM elements, which makes the system interesting in that both the VCI energies and the instanton TM elements are expected to make a significant contribution to the TSs in this system.

**Figure 10:**
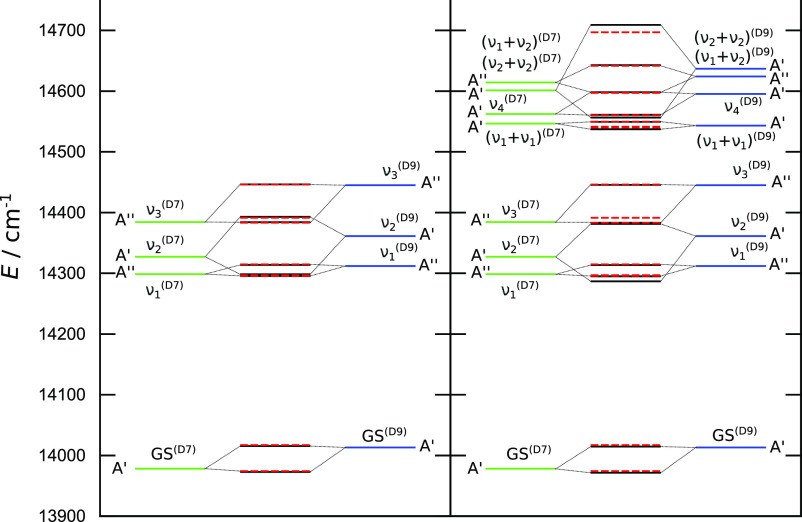
Vibrational tunneling spectrum of the lowest 8 (left panel) and 16 (right panel) states of PD malonaldehyde. Green and blue lines represent VCI energies of local wavefunctions in the D7 and D9 minima, respectively. Dashed red lines are obtained using a 2 × 2 TM model. Black lines in the left panel represent energies from an 8 × 8 TM model, and in the right panel, they represent energies from a 16 × 16 model. See the text for details.

In the PD malonaldehyde, the isotopic substitution causes a significant lowering of the zero-point energy, given in Table 5, from 14,682.45 to 13,978.19 cm^–1^ for the D7 minimum and to 14,013.04 cm^–1^ for the D9 minimum. Additionally, the excitation energies for the first seven excited states decrease as well, by up to 40 cm^–1^. As a result, the vibrational states are more closely spaced, see Figure 10, and larger interstate L/R mixings are expected.

**Table 5:** Harmonic and VCI Energies in Inverse Centimeters (cm^–1^) of the First Eight Local Vibrational States of PD Malonaldehyde Labeled by the Excited Normal Mode Frequencies^a^

	harmonic		VCI	
state	D7	D9	D7	D9
GS	14,228.18 (0.00)	14,253.67 (25.49)	13,978.19 (0.00)	14,013.04 (34.85)
ν_1_	14,492.55 (264.37)	14,492.10 (263.92)	14,298.70 (320.51)	14,311.75 (333.56)
ν_2_	14,522.65 (294.47)	14,547.57 (319.39)	14,327.02 (348.83)	14,361.38 (383.19)
ν_3_	14,568.85 (340.67)	14,626.56 (398.38)	14,384.49 (406.30)	14,444.96 (466.76)
ν_1_ + ν_1_	14,756.92 (528.74)	14,546.67 (502.34)	14,950.11 (568.48)	14,543.15 (564.96)
ν_4_	14,744.31 (516.13)	14,769.16 (540.98)	14,562.35 (584.16)	14,595.52 (617.32)
ν_2_ + ν_2_	14,817.13 (588.95)	14,841.47 (613.29)	14,601.27 (623.08)	14,637.01 (658.82)
ν_1_ + ν_2_	14,787.02 (558.84)	14,786.00 (557.82)	14,614.31 (636.12)	14,624.21 (646.02)

a Energies relative to the local ground state of the D7 minimum are given in parentheses.

The normal modes in PD malonaldehyde are qualitatively similar to the homoisotopic malonaldehyde, depicted in Figure 4. The ordering of local single-well states, labeled by the excited normal mode at the minimum, is also preserved upon deuteration, with the exception of the |(ν_1_ + ν_2_)^(D9)^⟩ and |(ν_2_ + ν_2_)^(D9)^|⟩ states, which exchange order. The TM elements, shown in Table 6, are remarkably similar to the homoisotopic malonaldehyde, which indicates that the wavefunctions in the barrier region are not significantly affected by the asymmetry. The error estimates due to the variation of the position of the dividing plane are shown in parentheses in Table 6 and are discussed in more detail in the Appendix.

**Table 6:** TM Elements Connecting the First Eight Local Vibrational States of Different Minima in PD Malonaldehyde in Inverse Centimeters (cm^–1^)^a^

	GS^(D9)^	ν_1_^(D9)^	ν_2_^(D9)^	ν_3_^(D9)^	(ν_1_ + ν_1_)^(D9)^	ν_4_^(D9)^	(ν_2_ + ν_2_)^(D9)^	(ν_1_ + ν_2_)^(D9)^
GS^(D7)^	–12.32 (0.005)	0.00 (0.00)	–21.95 (0.133)	0.00 (0.00)	–5.04 (0.042)	–4.63 (0.034)	–25.54 (0.343)	0.00 (0.00)
ν_1_^(D7)^	0.00 (0.00)	–6.86 (0.001)	0.00 (0.00)	6.52 (0.014)	0.00 (0.00)	0.00 (0.00)	0.00 (0.00)	–12.22 (0.077)
ν_2_^(D7)^	–21.89 (0.109)	0.00 (0.00)	–44.12 (0.031)	0.00 (0.00)	–8.95 (0.037)	–7.52 (0.029)	–55.89 (0.452)	0.00 (0.00)
ν_3_^(D7)^	0.00 (0.00)	7.37 (0.007)	0.00 (0.00)	8.64 (0.007)	0.00 (0.00)	0.00 (0.00)	0.00 (0.00)	13.14 (0.054)
(ν_1_ + ν_1_)^(D7)^	–5.16 (0.042)	0.00 (0.00)	–9.19 (0.034)	0.00 (0.00)	–4.58 (0.000)	–1.94 (0.001)	–9.45 (0.024)	0.00 (0.00)
ν_4_^(D7)^	–4.44 (0.029)	0.00 (0.00)	–7.25 (0.021)	0.00 (0.00)	–1.82 (0.001)	7.97 (0.004)	–7.84 (0.010)	0.00 (0.00)
(ν_2_ + ν_2_)^(D7)^	–25.42 (0.304)	0.00 (0.00)	–55.79 (0.349)	0.00 (0.00)	–9.18 (0.015)	–8.10 (0.001)	–75.67 (0.090)	0.00 (0.00)
(ν_1_ + ν_2_)^(D7)^	0.00 (0.00)	–12.19 (0.072)	0.00 (0.133)	11.59 (0.037)	0.00 (0.00)	0.00 (0.00)	0.00 (0.00)	–21.72 (0.006)

a Values in parentheses refer to the estimated error introduced by the neglect of the overlap between L/R local states, as explained in the Appendix.

We again consider pairwise interactions of the corresponding states in a 2 × 2 TM model. This is possible since the normal modes at both minima can approximately be mapped to one another using a symmetry operation. The pairs of states are no longer degenerate in this case, and the first-order perturbation theory cannot be used to estimate the TSs. Instead, the TS, obtained from the eigenvalues of the TM, is seen to be equal to the local energy difference corrected by the second-order perturbative terms
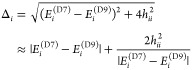
12where, in the last line of eq 12, we assumed that the TM element |*h*_*ii*_| ≪ |*E*_*i*_^(D7)^ – *E*_*i*_^(D9)^|. This assumption is certainly violated if there are other local states that are energetically close and coupled by the TM elements that are comparable in size.

The TM element for the GS is determined to be 12.32 cm^–1^ using the JFI method, which is in excellent agreement with the 12.4 cm^–1^ obtained by Jahr et al.^9^ using the RPI. The mixing angle for the GS was estimated experimentally by Baughcum et al.^10^ to be ϕ = 41° from the dipole moment measurements. The dipole moment of the D7/D9 isomer was modeled as a superposition of the dipole moments of D7 and D9 minima. These were, in turn, approximated by the dipole moments of D6D7D8 and D6D8D9 isomers, taken as two separate species, with the tunneling assumed to be suppressed. Reference (9) estimates the angle to be ϕ = 44° using local harmonic energies. Using VCI energies, we estimate the mixing angle to be ϕ = 35.3°, which indicates that the anharmonicity is indeed responsible for a decrease in its value, as speculated by Jahr et al.^9^ We were also able to estimate the effect of the inclusion of other local vibrational states on the mixing angle from the components of the TM eigenvectors in Table 8 as
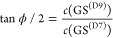
13which gives ϕ = 36.8°. It thus appears that the inclusion of additional interactions corrects the mixing angle toward the experimental value, which was obtained indirectly, as stated above, and may carry a considerable error.

Changes in the vibrational levels of the excited states in the 8 × 8 and the 16 × 16 matrix models, listed in Table 7, are qualitatively similar to the homoisotopic malonaldehyde due to the similarity in their TM elements. The vibrational tunneling spectrum is shown graphically in Figure 10. One significant difference here is that some doublet states change the order of their components after the inclusion of additional vibrational states in the model due to their proximity in energy after deuteration, as seen in Figure 10. Another difference is the absence of symmetry in the wavefunctions with respect to the symmetry operation that connects the minima in the homoisotopic case. As a result, the extensions of the 2 × 2 model to higher-dimensionality matrix models will mix both the lower and higher components of doublets, with all other doublet states. Finally, due to the proximity of vibrational states, the lower components of the (ν_1_ + ν_1_), ν_4_, and (ν_2_ + ν_2_) doublets are significantly mixed, as can be seen in Table 8. This mixing between the states changes their energies, but it is also expected to affect the intensity of the transition to the 11th state as its ν_4_ component (see Table 8) has a higher transition dipole moment, being the singly excited state.

**Table 7:** Vibrational Energy Levels of PD Malonaldehyde in Inverse Centimeters (cm^–1^) Obtained Using a Combined VCI/Instanton Approach^a^

no.	*E*^(pairs)^	*E*^(4)^	*E*^(8)^
1	13,974.27	13,972.91	13,971.48
2	14,016.96	14,015.52	14,014.49
3	14,296.86	14,298.42	14,286.79
4	14,295.75	14,295.44	14,294.91
5	14,314.69	14,313.95	14,313.54
6	14,391.55	14,392.78	14,381.17
7	14,383.28	14,384.05	14,383.32
8	14,446.16	14,446.45	14,445.64
9	14,540.00		14,537.31
10	14,549.82		14,549.32
11	14,541.38		14,556.57
12	14,560.53		14,561.08
13	14,597.33		14,598.21
14	14,596.99		14,598.33
15	14,641.54		14,642.66
16	14,696.90		14,709.18

a *E*^(pairs)^, *E*^(4)^, and *E*^(8)^ are energies obtained from the 2 × 2, 8 × 8, and 16 × 16 matrix models, respectively, as explained in the text.

**Table 8:**
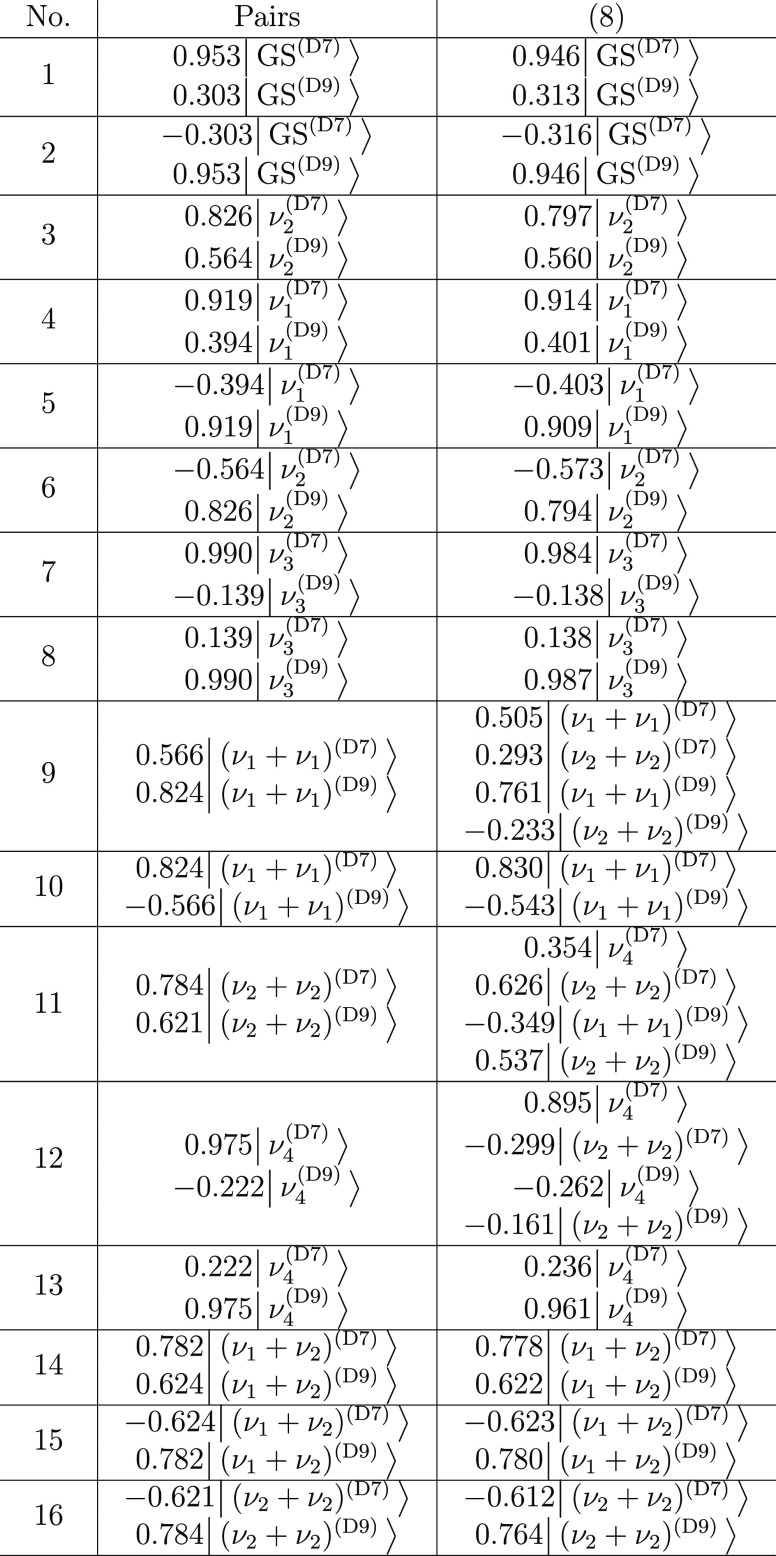
Dominant Configurations of Vibrational States of PD Malonaldehyde, Obtained as the Eigenvectors of the TM in the 2 × 2 (Pairs) and 16 × 16 (Eight-State) Models, as Described in the Text

## 4 Conclusions

We applied a combination of VCI and the instanton theory to calculate vibrational tunneling spectra of some exemplary double-well systems in full dimensionality at a much reduced computational cost in comparison with the exact QM methods. The VCI method was used to compute the single-well vibrational spectra, while the recently developed instanton method was used to determine the wavefunctions inside the barrier that separates the wells at a comparatively negligible computational cost. The interaction between the states of different wells was obtained from the Herring formula evaluated at a dividing surface inside the barrier. The Herring formula was rederived in an extended *N* × *N* matrix model (*N* > 2) , and the size of the associated leading error term was analyzed.

The accuracy of our approach was first tested on a model 2D system. It was shown that the JFI method can be used to compute TM elements that connect states in inequivalent wells and that have excitations in different normal modes. The energy levels of an asymmetric system exhibit avoided crossings with the variation of frequency or depth of one well relative to that of the other. The VCI calculation of local energies proved to be necessary in order to reproduce the exact QM results with high accuracy. The method was then tested on malonadehyde in full dimensionality, where good agreement was achieved with the exact MCTDH results in the absolute energies and the splittings. It was shown that the extension of the standard 2 × 2 model to include more states can influence the vibrational energies. The results are not affected dramatically in the case of malonaldehyde, but it was shown that the model is able to accommodate the additional vibrational states in the systems where they lie close in energy.

Finally, the method was used to calculate the vibrational spectrum of the low-lying states in PD malonaldehyde, which is near the computational limit of the presently available exact QM methods. The ground-state mixing angle was compared to the experimental value, and the influence of including additional vibrational states was shown to affect the angle and the order of some states in the spectrum.

The method is expected to perform well for mid-sized molecules, where rotational motion, which is neglected in this work, can be separated from the tunneling dynamics, and for moderately anharmonic systems with high barriers and, consequently, small TSs. It is exactly in these circumstances that the exact QM methods come at a prohibitive computational cost. The developed combined approach can be used to calculate the low-lying vibrational spectra in systems with an arbitrary number of wells, which are not necessarily related by symmetry. This makes the method particularly suitable to the studies of clusters, for example, for the assignment of spectra in water clusters, which feature multiple minima and high barriers in their bifurcation dynamics (where hydrogen bonds are broken and reformed). The computational cost of our approach is concentrated in solving the single-well spectra separately. The instanton theory can also be combined with other high-level methods, instead of VCI, and the combined, dual-level, approach can be used to calculate the tunneling spectra in general multidimensional asymmetric well systems, beyond molecular applications and chemistry.

## Appendix

### Dependence of TM Elements on the Position of the Dividing Plane

TM elements between the states with, at most, one excitation (ν, ν′ = 0–1) can be expressed as

14

where the contribution of **U** terms in eq 9 has been neglected. It is known that unless these terms exclusively contribute to the TM elements, they introduce the dependence of the TM element *h*_νν′_ on the connection point.^42^ Therefore, we omit them from the treatment here to separate the effect of **U** terms from the effect of asymmetry in the dependence of the TM element with the variation of the connection point *S*_cp_. The case of multiple excitations, as well as that of the **U** terms, can be derived in an analogous fashion.

Dependence on the position of the connection point can be determined by differentiating the TM element in eq 14 with respect to *S*_cp_. Small changes in the connection point correspond to the addition of function *f*(*S*) of the following form

15

to the potential in the action integral, where ε denotes the change in the connection point. As discussed in Section 1 of the Supporting Information, the addition of *f* in eq 15 does not change the shape of the path for *d* ≪ *V*(*S*_cp_). The differentiation can thus be performed on the same path. Furthermore, if the connection point is located deep inside the barrier, we can approximate

16

Following the differentiation, as described in ref (42), we obtain

17

where *E*^(L/R)^ are the energies of the local L/R wavefunctions and **A**_⊥_ is the matrix **A** projected onto the space orthogonal to the MAP. From eq 17, it is evident that the TM element will only be invariant with respect to variation in *S*_cp_ if the two local L/R states are in resonance. Otherwise, the TM element depends on the position of the connection point, and the dependence is stronger for a larger mismatch in the local energies.

The dependence on the connection point can be traced to the neglected integral in the derivation of the Herring formula in eq 3 involving the ϕ_ν_^(L)^ϕ_ν′_^(R)^ term. Inclusion of this term transforms the formula for the TM element into

18

where the second integral is taken over the space on the “left” side of the dividing plane. We then differentiate the extended expression, eq 18, with respect to *S*_cp_ and observe that the derivative of the additional term in eq 18 is the integral over the dividing plane. The derivative of the TM element becomes

19

where use has been made of eq 17. We note now that the surface integral in eq 19 differs from the “old version” of *h*_νν′_ only by the momentum term *p̅*_0_. However, this factor is taken to be constant on the dividing plane, and it can be taken out of the integral, which leads to

20

While it would seem obvious to use the instanton wavefunctions in eq 6 and compute the additional term that arises in eq 18 in order to eliminate the dependence on *S*_cp_, the semiclassical approximation breaks down beyond the barrier region and would lead to a divergence of the integral. We are thus left with no option but to neglect the term and treat it as a source of error in the TM element.

We tried to estimate the size of the error term, introduced above, by calculating the overlap of the harmonic oscillator wavefunctions centered at the two minima. The overlap is obtained following the method of ref (64), where it was used to calculate Frank–Condon factors between the shifted harmonic oscillators. The calculation requires the knowledge of molecular geometries and harmonic frequencies in the two minima and does not add to the overall computational cost. The errors for PD malonaldehyde were found to be small and are listed in the parentheses in Table 6. We found that it was safe to neglect the error terms unless the connection point is moved far from the position of the barrier top or unless the energy difference between the two states is large in relation to the barrier height. However, we note that as the energy difference increases, the contribution of the TM element to the energy level decreases, as seen from the second-order perturbation treatment

21

Since *h*_*ij*_ are expected to be small in the deep tunneling regime, the contributions of the states that lie far from the resonance quickly approach zero. Thus, even for larger energy differences, the computed energy levels remain stable.

Dependence of the TM elements on the position of the dividing plane was also tested by moving the dividing plane from 0.25*S*_tot_ to 0.75*S*_tot_, where *S*_tot_ is the MAP length. All the matrix elements displayed monotonous dependence on *S*_cp_, with an inflection point located near the position of the barrier top and a region of relative stability around it. As the dividing plane is moved away from the barrier top, some TM elements begin to change significantly. These TM elements connect the states with a significant disparity in their local energies. For example, the TM elements between the (ν_2_ + ν_2_) state and the GS or the ν_2_-excited state change from −11 to −55 cm^–1^ and from −36 to −80 cm^–1^, respectively. However, a large energy difference between the involved states also implies that the TM elements do not profoundly affect the overall energy. The GS TS varies by ≈7 cm^–1^ in this region. If we limit ourselves to the region between 0.4*S*_tot_ and 0.6*S*_tot_, the variation of the GS TS is only 1.5 cm^–1^, which is within the error of the VCI method used to compute the local energies. Similar errors are observed for the other energy levels. The method thus gives the best results when the dividing plane is placed in the vicinity of the barrier top.

## Supporting Information Available

MAP invariance with respect to the addition of a switching function; VSCF and VCI; dependence of vibrational energies of the lowest six states in the double-well potential on ω_1_^(R)^, ω_2_^(R)^, and *d*; and dependence of vibrational energies and TSs in the 2D model potential on ω_1_^(R)^, ω_2_^(R)^, and *d* (PDF)

## References

[ref1] Bell R. P. The Tunnel Effect in Chemistry; Chapman and Hall: London, 1980.

[ref2] Hund F. Zur Deutung der Molekelspektren. III. Z. Phys. 1927, 43, 805–826. 10.1007/bf01397249.

[ref3] Benderskii V. A.; Makarov D. E.; Wight C. A. Chemical Dynamics at Low Temperatures; Advances in Chemical Physics; Wiley: New York, 1994; Vol. 88.

[ref4] Urban Š.; Špirko V.; Papoušek D.; Kauppinen J.; Belov S. P.; Gershtein L. I.; Krupnov A. F. A simultaneous analysis of the microwave, submillimeterwave, far infrared, and infrared-microwave two-photon transitions between the ground and ν2 inversion-rotation levels of 14NH3. J. Mol. Spectrosc. 1981, 88, 274–292. 10.1016/0022-2852(81)90179-x.

[ref5] Firth D. W.; Beyer K.; Dvorak M. A.; Reeve S. W.; Grushow A.; Leopold K. R. Tunable far-infrared spectroscopy of malonaldehyde. J. Chem. Phys. 1991, 94, 1812 10.1063/1.459955.

[ref6] Mengesha E. T.; Sepioł J.; Borowicz P.; Waluk J. Vibrations of porphycene in the S 0 and S 1 electronic states: Single vibronic level dispersed fluorescence study in a supersonic jet. J. Chem. Phys. 2013, 138, 174201 10.1063/1.4802769. 23656125

[ref7] Tanaka K.; Toshimitsu M.; Harada K.; Tanaka T. Determination of the proton tunneling splitting of the vinyl radical in the ground state by millimeter-wave spectroscopy combined with supersonic jet expansion and ultraviolet photolysis. J. Chem. Phys. 2004, 120, 3604–3618. 10.1063/1.1642583. 15268522

[ref8] Cvitaš M. T.; Richardson J. O. Molecular Spectroscopy and Quantum Dynamics; Marquardt R., Quack M., Eds.; Elsevier, 2020; Chapter 9, pp 301–326.

[ref9] Jahr E.; Laude G.; Richardson J. O. Instanton theory of tunneling in molecules with asymmetric isotopic substitutions. J. Chem. Phys. 2020, 153, 094101 10.1063/5.0021831. 32891112

[ref10] Baughcum S. L.; Duerst R. W.; Rowe W. F.; Smith Z.; Wilson E. B. Microwave spectroscopic study of malonaldehyde (3-hydroxy-2-propenal). 2. Structure, dipole moment, and tunneling. J. Am. Chem. Soc. 1981, 103, 6296–6303. 10.1021/ja00411a005.

[ref11] Zhang D. H.; Wu Q.; Zhang J. Z. H.; von Dirke M.; Bačić Z. Exact full-dimensional bound state calculations for (HF) 2 , (DF) 2 , and HFDF. J. Chem. Phys. 1995, 102, 2315–2325. 10.1063/1.468719.

[ref12] Šmydke J.; Fábri C.; Sarka J.; Császár A. G. Rovibrational quantum dynamics of the vinyl radical and its deuterated isotopologues. Phys. Chem. Chem. Phys. 2019, 21, 3453–3472. 10.1039/c8cp04672g. 30406229

[ref13] Liu K.; Brown M. G.; Viant M. R.; Cruzan J. D.; Saykally R. J. Far infrared VRT spectroscopy of two water trimer isotopomers: Vibrationally averaged structures and rearrangement dynamics. Mol. Phys. 1996, 89, 1373–1396. 10.1080/00268979609482547. 11539422

[ref14] Eraković M.; Cvitaš M. T. Tunnelling splitting patterns in some partially deuterated water trimers. Phys. Chem. Chem. Phys. 2021, 23, 4240–4254. 10.1039/d0cp06135b. 33586727

[ref15] González L.; Mó O.; Yáñez M. High-Level ab Initio Calculations on the Intramolecular Hydrogen Bond in Thiomalonaldehyde. J. Phys. Chem. A 1997, 101, 9710–9719. 10.1021/jp970735z.

[ref16] Bondybey V. E.; Haddon R. C.; Rentzepis P. M. Spectroscopy and dynamics of 9-hydroxyphenalenone and of its 5-methyl derivative in solid neon: effect of methyl group upon vibrational relaxation. J. Am. Chem. Soc. 1984, 106, 5969–5973. 10.1021/ja00332a036.

[ref17] Oppenländer A.; Rambaud C.; Trommsdorff H. P.; Vial J.-C. Translational tunneling of protons in benzoic-acid crystals. Phys. Rev. Lett. 1989, 63, 1432–1435. 10.1103/physrevlett.63.1432. 10040566

[ref18] Hall B. V.; Whitlock S.; Anderson R.; Hannaford P.; Sidorov A. I. Condensate Splitting in an Asymmetric Double Well for Atom Chip Based Sensors. Phys. Rev. Lett. 2007, 98, 030402 10.1103/PhysRevLett.98.030402. 17358663

[ref19] Takahashi S.; Tupitsyn I. S.; van Tol J.; Beedle C. C.; Hendrickson D. N.; Stamp P. C. E. Decoherence in crystals of quantum molecular magnets. Nature 2011, 476, 76–79. 10.1038/nature10314. 21775988

[ref20] Johnson P. R.; Parsons W. T.; Strauch F. W.; Anderson J. R.; Dragt A. J.; Lobb C. J.; Wellstood F. C. Macroscopic Tunnel Splittings in Superconducting Phase Qubits. Phys. Rev. Lett. 2005, 94, 187004 10.1103/physrevlett.94.187004. 15904404

[ref21] Felker P. M.; Bačić Z. Weakly bound molecular dimers: Intramolecular vibrational fundamentals, overtones, and tunneling splittings from full-dimensional quantum calculations using compact contracted bases of intramolecular and low-energy rigid-monomer intermolecular eigenstates. J. Chem. Phys. 2019, 151, 024305 10.1063/1.5111131. 31301718

[ref22] Schröder M.; Meyer H.-D. Calculation of the vibrational excited states of malonaldehyde and their tunneling splitting with the multi-configuration time-dependent Hartree method. J. Chem. Phys. 2014, 141, 034116 10.1063/1.4890116. 25053310

[ref23] Hammer T.; Manthe U. Iterative diagonalization in the state-averaged multi-configurational time-dependent Hartree approach: Excited state tunneling splittings in malonaldehyde. J. Chem. Phys. 2012, 136, 054105 10.1063/1.3681166. 22320723

[ref24] Leforestier C.; Szalewicz K.; van der Avoird A. Spectra of water dimer from a new ab initio potential with flexible monomers. J. Chem. Phys. 2012, 137, 014305 10.1063/1.4722338. 22779646

[ref25] Vaillant C. L.; Wales D. J.; Althorpe S. C. Tunneling splittings from path-integral molecular dynamics using a Langevin thermostat. J. Chem. Phys. 2018, 148, 234102 10.1063/1.5029258. 29935506

[ref26] Vaillant C. L.; Wales D. J.; Althorpe S. C. Tunneling Splittings in Water Clusters from Path Integral Molecular Dynamics. J. Phys. Chem. Lett. 2019, 10, 7300–7304. 10.1021/acs.jpclett.9b02951. 31682130

[ref27] Benderskii V. A.; Vetoshkin E. V.; Grebenshchikov S. Y.; von Laue L.; Trommsdorff H. P. Tunneling splitting in vibrational spectra of non-rigid molecules. I. Perturbative instanton approach. Chem. Phys. 1997, 219, 119–142. 10.1016/s0301-0104(97)00118-3.

[ref28] Smedarchina Z.; Siebrand W.; Fernández-Ramos A. The rainbow instanton method: A new approach to tunneling splitting in polyatomics. J. Chem. Phys. 2012, 137, 224105 10.1063/1.4769198. 23248985

[ref29] Richardson J. O.; Althorpe S. C. Ring-polymer instanton method for calculating tunneling splittings. J. Chem. Phys. 2011, 134, 054109 10.1063/1.3530589. 21303094

[ref30] Mil’nikov G. V.; Nakamura H. Practical implementation of the instanton theory for the ground-state tunneling splitting. J. Chem. Phys. 2001, 115, 6881–6897. 10.1063/1.1406532.

[ref31] Eraković M.; Vaillant C. L.; Cvitaš M. T. Instanton theory of ground-state tunneling splittings with general paths. J. Chem. Phys. 2020, 152, 084111 10.1063/1.5145278. 32113369

[ref32] Cvitaš M. T.; Althorpe S. C. Locating instantons in calculations of tunneling splittings: The test case of malonaldehyde. J. Chem. Theory Comput. 2016, 12, 787–803. 10.1021/acs.jctc.5b01073. 26756608

[ref33] Cvitaš M. T. Quadratic string method for locating instantons in tunneling splitting calculations. J. Chem. Theory Comput. 2018, 14, 1487–1500. 10.1021/acs.jctc.7b00881. 29360359

[ref34] Mil’nikov G. V.; Ishida T.; Nakamura H. Tunneling Splitting of Energy Levels and Rotational Constants in the Vinyl Radical C_2_H_3_. J. Phys. Chem. A 2006, 110, 5430–5435. 10.1021/jp055667s. 16623471

[ref35] Mil’nikov G.; Kühn O.; Nakamura H. Ground-state and vibrationally assisted tunneling in the formic acid dimer. J. Chem. Phys. 2005, 123, 074308 10.1063/1.2000257. 16229571

[ref36] Vaillant C. L.; Cvitaš M. T. Rotation-tunneling spectrum of the water dimer from instanton theory. Phys. Chem. Chem. Phys. 2018, 20, 26809–26813. 10.1039/c8cp04991b. 30328431

[ref37] Zwart E.; ter Meulen J. J.; Leo Meerts W.; Coudert L. H. The submillimeter rotation tunneling spectrum of the water dimer. J. Mol. Spectrosc. 1991, 147, 27–39. 10.1016/0022-2852(91)90165-7.

[ref38] Keutsch F. N.; Cruzan J. D.; Saykally R. J. The water trimer. Chem. Rev. 2003, 103, 2533–2578. 10.1021/cr980125a. 12848579

[ref39] Richardson J. O.; Pérez C.; Lobsiger S.; Reid A. A.; Temelso B.; Shields G. C.; Kisiel Z.; Wales D. J.; Pate B. H.; Althorpe S. C. Concerted Hydrogen-Bond Breaking by Quantum Tunneling in the Water Hexamer Prism. Science 2016, 351, 1310–1313. 10.1126/science.aae0012. 26989250

[ref40] Richardson J. O.; Wales D. J.; Althorpe S. C.; McLaughlin R. P.; Viant M. R.; Shih O.; Saykally R. J. Investigation of Terahertz Vibration-Rotation Tunneling Spectra for the Water Octamer. J. Phys. Chem. A 2013, 117, 6960–6966. 10.1021/jp311306a. 23286830

[ref41] Cvitaš M. T.; Richardson J. O. Quantum Tunnelling Pathways of the Water Pentamer. Phys. Chem. Chem. Phys. 2019, 22, 1035–1044. 10.1039/c9cp05561d. 31859328

[ref42] Eraković M.; Cvitaš M. T. Tunneling splittings of vibrationally excited states using general instanton paths. J. Chem. Phys. 2020, 153, 134106 10.1063/5.0024210. 33032414

[ref43] Mil’nikov G. V.; Nakamura H. Instanton theory for the tunneling splitting of low vibrationally excited states. J. Chem. Phys. 2005, 122, 124311 10.1063/1.1869989. 15836382

[ref44] Garg A. Tunnel splittings for one-dimensional potential wells revisited. Am. J. Phys. 2000, 68, 430–437. 10.1119/1.19458.

[ref45] Herring C. Critique of the Heitler-London Method of Calculating Spin Couplings at Large Distances. Rev. Mod. Phys. 1962, 34, 631–645. 10.1103/revmodphys.34.631.

[ref46] Cesi F.; Rossi G. C.; Testa M. Non-symmetric double well and euclidean functional integral. Ann. Phys. 1991, 206, 318–333. 10.1016/0003-4916(91)90003-q.

[ref47] Mugnai D.; Ranfagni A. A simple approaach to quantum fluctuations in tunneling processes. Phys. Lett. A 1985, 109, 219–223. 10.1016/0375-9601(85)90307-x.

[ref48] Leggett A. J.; Chakravarty S.; Dorsey A. T.; Fisher M. P. A.; Garg A.; Zwerger W. Dynamics of the dissipative two-state system. Rev. Mod. Phys. 1987, 59, 1–85. 10.1103/revmodphys.59.1.

[ref49] Dekker H. Quantum mechanical barrier problems: I. Coherence and tunnelling in asymmetric potentials. Phys. A 1987, 146, 375–386. 10.1016/0378-4371(87)90274-3.

[ref50] Song D.-Y. Tunneling and energy splitting in an asymmetric double-well potential. Ann. Phys. 2008, 323, 2991–2999. 10.1016/j.aop.2008.09.004.

[ref51] Song D.-Y. Localization or tunneling in asymmetric double-well potentials. Ann. Phys. 2015, 362, 609–620. 10.1016/j.aop.2015.08.029.

[ref52] Halataei S. M. H.; Leggett A. J. Tunnel splitting in asymmetric double well potentials: an improved WKB calculation. 2017, arXiv preprint arXiv: 1703.05758

[ref53] Siebrand W.; Smedarchina Z.; Fernández-Ramos A. Communication: Selection rules for tunneling splitting of vibrationally excited levels. J. Chem. Phys. 2013, 139, 021101 10.1063/1.4813002. 23862915

[ref54] Benderskii V. A.; Vetoshkin E. V.; Trommsdorff H. P. Tunneling splittings in vibrational spectra of non-rigid molecules: VI. Asymmetric double-well potentials. Chem. Phys. 1999, 244, 299–317. 10.1016/s0301-0104(99)00143-3.

[ref55] Benderskii V. A.; Vetoshkin E. V.; Irgibaeva I. S.; Trommsdorff H. P. Tunneling splittings in vibrational spectra of non-rigid molecules IX. Malonaldehyde and its isotopomers as a test case for fully coupled multidimensional tunneling dynamics. Chem. Phys. 2000, 262, 393–422. 10.1016/s0301-0104(00)00319-0.

[ref56] Carter S.; Bowman J. M.; Handy N. C. Extensions and tests of ”multimode”: a code to obtain accurate vibration/rotation energies of many-mode molecules. Theor. Chim. Acta 1998, 100, 191–198. 10.1007/s002140050379.

[ref57] Christoffel K. M.; Bowman J. M. Investigations of self-consistent field, scf ci and virtual stateconfiguration interaction vibrational energies for a model three-mode system. Chem. Phys. Lett. 1982, 85, 220–224. 10.1016/0009-2614(82)80335-7.

[ref58] Bowman J. M.; Christoffel K.; Tobin F. Application of SCF-SI theory to vibrational motion in polyatomic molecules. J. Phys. Chem. 1979, 83, 905–912. 10.1021/j100471a005.

[ref59] Bowman J. M.; Carter S.; Huang X. MULTIMODE: A code to calculate rovibrational energies of polyatomic molecules. Int. Rev. Phys. Chem. 2003, 22, 533–549. 10.1080/0144235031000124163.

[ref60] Rauhut G. Efficient calculation of potential energy surfaces for the generation of vibrational wave functions. J. Chem. Phys. 2004, 121, 9313–9322. 10.1063/1.1804174. 15538851

[ref61] Wang Y.; Braams B. J.; Bowman J. M.; Carter S.; Tew D. P. Full-dimensional quantum calculations of ground-state tunneling splitting of malonaldehyde using an accurate ab initio potential energy surface. J. Chem. Phys. 2008, 128, 224314 10.1063/1.2937732. 18554020

[ref62] Ferro-Costas D.; Fernández-Ramos A. Tunnelling in Molecules: Nuclear Quantum Effects from Bio to Physical Chemistry; Kästner J., Kozuch S., Eds.; Royal Society of Chemistry, 2020; Chapter 9, pp 283–327. 10.1039/9781839160370-00283

[ref63] Lüttschwager N. O. B.; Wassermann T. N.; Coussan S.; Suhm M. A. Vibrational tuning of the Hydrogen transfer in malonaldehyde – a combined FTIR and Raman jet study. Mol. Phys. 2013, 111, 2211–2227. 10.1080/00268976.2013.798042.

[ref64] Berger R.; Fischer C.; Klessinger M. Calculation of the Vibronic Fine Structure in Electronic Spectra at Higher Temperatures. 1. Benzene and Pyrazine. J. Phys. Chem. A 1998, 102, 7157–7167. 10.1021/jp981597w.

