# Supplementary material for: Vibrational Tunneling Spectra of Molecules with Asymmetric Wells: A Combined Vibrational Configuration Interaction and Instanton Approach
Source: J Chem Theory Comput. 2022 Apr 19;18(5):2785–802. doi: 10.1021/acs.jctc.2c00124 (PMC9097297; doi:10.1021/acs.jctc.2c00124)
Supplement: Supplementary file 1 — ct2c00124_si_001.pdf [file ct2c00124_si_001.pdf]

Supporting Information: Vibrational tunneling spectra of molecules with asymmetric wells: a combined vibrational configuration interaction and instanton approach

Mihael Eraković[†] and Marko T. Cvitaš[*,‡]

[†]*Department of Physical Chemistry, Ruđer Bošković Institute, Bijenička Cesta 54, 10000 Zagreb, Croatia*

[‡]*Department of Physics, Faculty of Science, University of Zagreb, Bijenička Cesta 32, 10000 Zagreb, Croatia*

E-mail: mcvitas@phy.hr

1 MAP invariance with respect to the addition of a switching function

We show below that the addition of a function $f(S)$, which only depends on the coordinate S along the path, to the potential,

$$\tilde{V}(\mathbf{x}) = V(\mathbf{x}) - f(S), \tag{1}$$

does not change the shape of the MAP, but only scales the imaginary time parameter τ. The statement is valid if the function f does not change the position or shape of the minima, i.e., it satisfies

$$\begin{aligned}\lim_{S\to 0(S_{\rm tot})} f(S) &= 0,\\ \lim_{S\to 0(S_{\rm tot})} f'(S) &= 0,\\ \lim_{S\to 0(S_{\rm tot})} f''(S) &= 0,\end{aligned} \tag{2}$$

and is negligible in comparison with the potential in the region between the minima, $f(S) \ll V(S)$.

Path $\mathbf{x}(\tau)$ is the characteristic for potential V and it satisfies

$$\begin{aligned}\frac{d^2}{d\tau^2}\mathbf{x}(\tau) &= \nabla V.\\ \frac{dS}{d\tau} &= p_0 = \sqrt{2V},\end{aligned} \tag{3}$$

where S is the arc length distance along the path. We define the scaled imaginary time as

$$d\tilde{\tau} = \frac{d\tau}{\sqrt{1-\frac{f(S)}{V}}}. \tag{4}$$

The scaled momentum then becomes

$$\tilde{p}_0 = \frac{dS}{d\tilde{\tau}} = \sqrt{2(V-f(S))} = \sqrt{2\tilde{V}}. \tag{5}$$

The momentum vector transforms as

$$\begin{aligned}\frac{d}{d\tilde{\tau}}\mathbf{x}(\tilde{\tau}) &= \frac{d\mathbf{x}}{d\tau}\sqrt{1-\frac{f(S)}{V}},\\ \tilde{\mathbf{p}}_0 &= \mathbf{p}_0\sqrt{1-\frac{f(S)}{V}},\end{aligned} \tag{6}$$

whereas the acceleration becomes

$$\frac{d^2}{d\tilde{\tau}^2}\mathbf{x}(\tilde{\tau}) = \nabla(V - f(S)) + \frac{f(S)}{V}\left(\frac{\mathbf{p}_0}{p_0}\frac{d}{dS}V - \nabla V\right),$$
$$\frac{d^2}{d\tilde{\tau}^2}\mathbf{x}(\tilde{\tau}) = \nabla\tilde{V} - \frac{f(S)}{V}\left(\nabla V\right)_{\perp}, \tag{7}$$

where the symbol $\perp$ in the subscript denotes the component of the vector that is perpendicular to the path. Now, if the function $f(S)$ satisfies the conditions in Eq. (2) and is significantly smaller than the potential, the second term on the right hand side of Eq. (7) is small everywhere on the path in comparison to the gradient of the potential and can be ignored. Eq. (7) then takes the form of the equation of characteristic, but on the modified potential $\tilde{V}$.

2 Vibrational self consistent field (VSCF) and vibrational configuration interaction (VCI)

The idea behind the VSCF approach is to approximate the vibrational wavefunction by a Hartree product of single-mode (1M) functions as

$$\psi(\mathbf{q}) = \phi_0^{(1)}(q_1)\dots\phi_0^{(N)}(q_N), \tag{8}$$

where q_i is the i-th normal mode coordinate. The above form can efficiently be employed in combination with the n-mode representation[S1,S2] of the potential

$$V(\mathbf{q}) = V_{\min} + \sum_{i=1}^{N} V_i^{(1\mathrm{M})}(q_i) + \sum_{i=1}^{N-1}\sum_{j=i}^{N} V_{ij}^{(2\mathrm{M})}(q_i, q_j) + \dots \tag{9}$$

In this paper, the expansion was truncated at the two-mode (2M) terms. Using wavefunction in Eq. (8) and variational principle, it can be shown[S3–S5] that the optimal 1M functions satisfy a set of coupled 1M equations

$$\frac{1}{2}\frac{d^2}{dq_i^2}\phi^{(i)}(q_i) + (V_{\min} + V_i^{(1\mathrm{M})}(q_i) +$$
$$\sum_{\substack{j=1\\ j\neq i}}^{N_{\mathrm{dof}}} \left\langle \phi_0^{(j)}(q_j)\left|V_{ij}^{(2\mathrm{M})}(q_i, q_j)\right|\phi_0^{(j)}(q_j)\right\rangle)\phi^{(i)}(q_i) = \varepsilon_i\phi^{(i)}(q_i), \tag{10}$$

which can be solved via an SCF algorithm. The 1M functions ϕ_0^i are expanded in a basis set $\{\chi_j^{(i)}\}$. In this paper, the lowest N_{basis} states of the harmonic oscillator (HO) are used. The

1M and 2M potential energy terms in Eq. (9) are fitted to polynomials of order N_{fit} as

$$V_i^{(1\text{M})}(q_i) = \sum_{j=2}^{N_{\text{fit}}} C_j^{(i)} q_i^j$$

$$V_{ij}^{(2\text{M})}(q_i, q_j) = \sum_{k=1}^{N_{\text{fit}}-1} \sum_{l=1}^{N_{\text{fit}}-k} C_{kl}^{(ij)} q_i^k q_j^l. \tag{11}$$

These choices enable us to compute the potential matrix elements exactly. For that purpose, we calculate the matrices $\mathbf{Q}_i^{(j)}$, that represent operators q_i^j in the HO basis. This can be done recursively using ladder operators

$$\langle\, \chi_j^{(i)} \,|q_i^n|\, \chi_k^{(i)} \,\rangle = \frac{1}{\sqrt{2\omega_i}} \langle\, \chi_j^{(i)} \,|q_i^{n-1}(\hat{a}_i + \hat{a}_i^\dagger)|\, \chi_k^{(i)} \,\rangle =$$

$$= \sqrt{\frac{k}{2\omega_i}} \langle\, \chi_j^{(i)} \,|q_i^{n-1}|\, \chi_{k-1}^{(i)} \,\rangle + \sqrt{\frac{k+1}{2\omega_i}} \langle\, \chi_j^{(i)} \,|q_i^{n-1}|\, \chi_{k+1}^{(i)} \,\rangle. \tag{12}$$

The matrices $\mathbf{Q}_i^{(j)}$ are then stored and the SCF algorithm is started using the initial guess $\phi_0^{(i)} = \chi_0^{(i)}$. The effective Hamiltonian in Eq. (10) is constructed for each mode in the HO basis, diagonalized and the obtained 1M functions corresponding to the lowest eigenvalue taken as new $\phi_0^{(i)}$. Once VSCF has converged, the obtained virtual 1M functions are used to construct the VCI Hamiltonian

$$H_{IJ} = \langle\, \phi_{i_1}^{(1)} \dots \phi_{i_N}^{(N)} \,|\hat{H}|\, \phi_{j_1}^{(1)} \dots \phi_{j_N}^{(N)} \,\rangle. \tag{13}$$

The matrix elements of H_{IJ} are computed using $\mathbf{Q}_i^{(j)}$ matrices and the coefficients of 1M functions in the HO basis. Its eigenvalues represent VCI energies, while the eigenvectors can be used to determine the dominant configuration $\phi_{i_1}^{(1)} \dots \phi_{i_N}^{(N)}$ and tell us which normal modes are excited in the particular state.

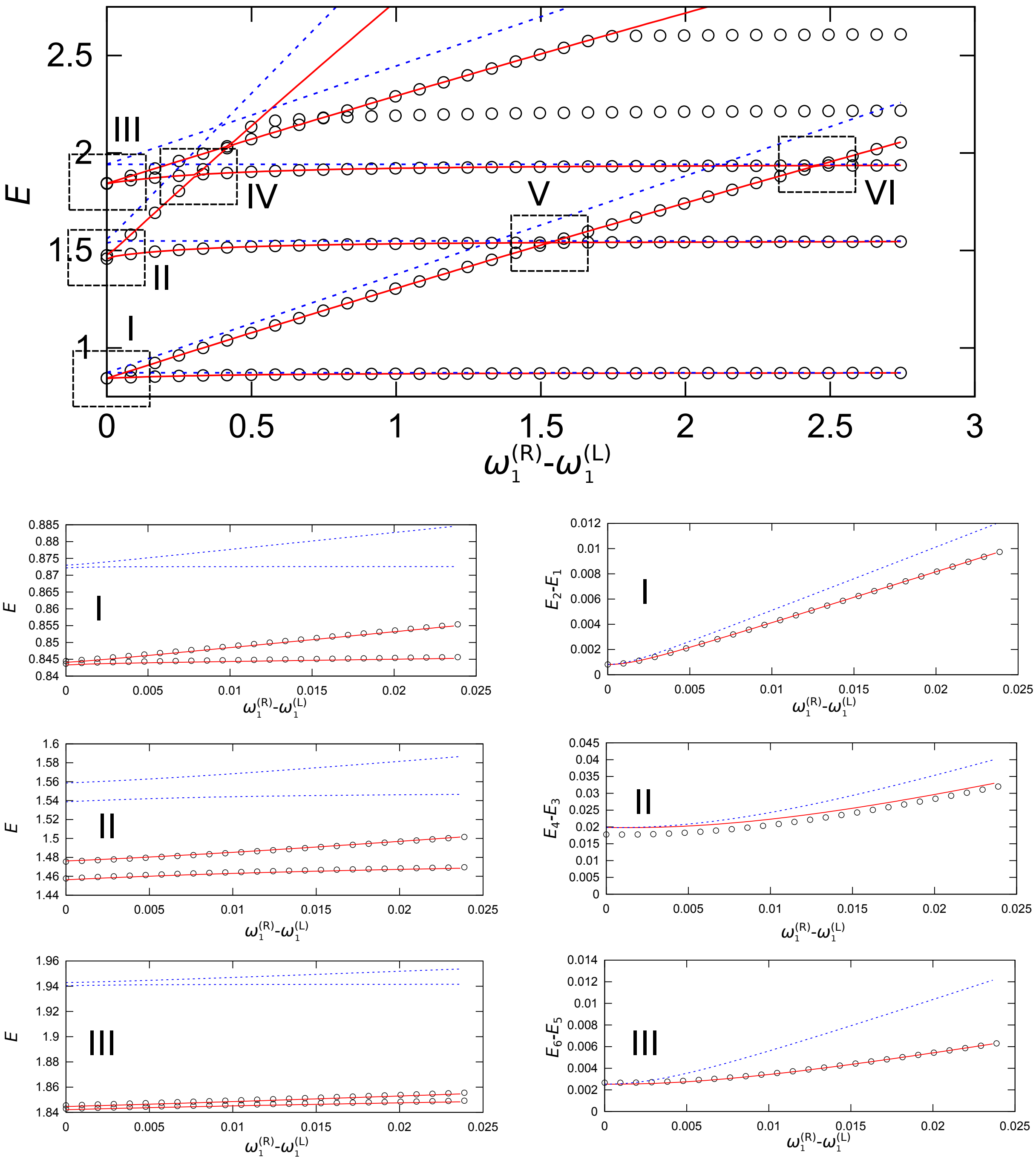

Figure S1: Dependence of vibrational energies of the lowest 6 states in the double-well potential given by Eq. (10) (see accompanying article) on $\omega_1^{(R)}$. Circles represent quantum-mechanical values, blue lines are obtained using instanton method with harmonic energies, red lines are obtained using a combined VCI/instanton approach. Frames I-III in the top panel are shown magnified in the left column panels below, and the dependence of the associated tunneling splittings on $\omega_1^{(R)}$ in the right column panels below.

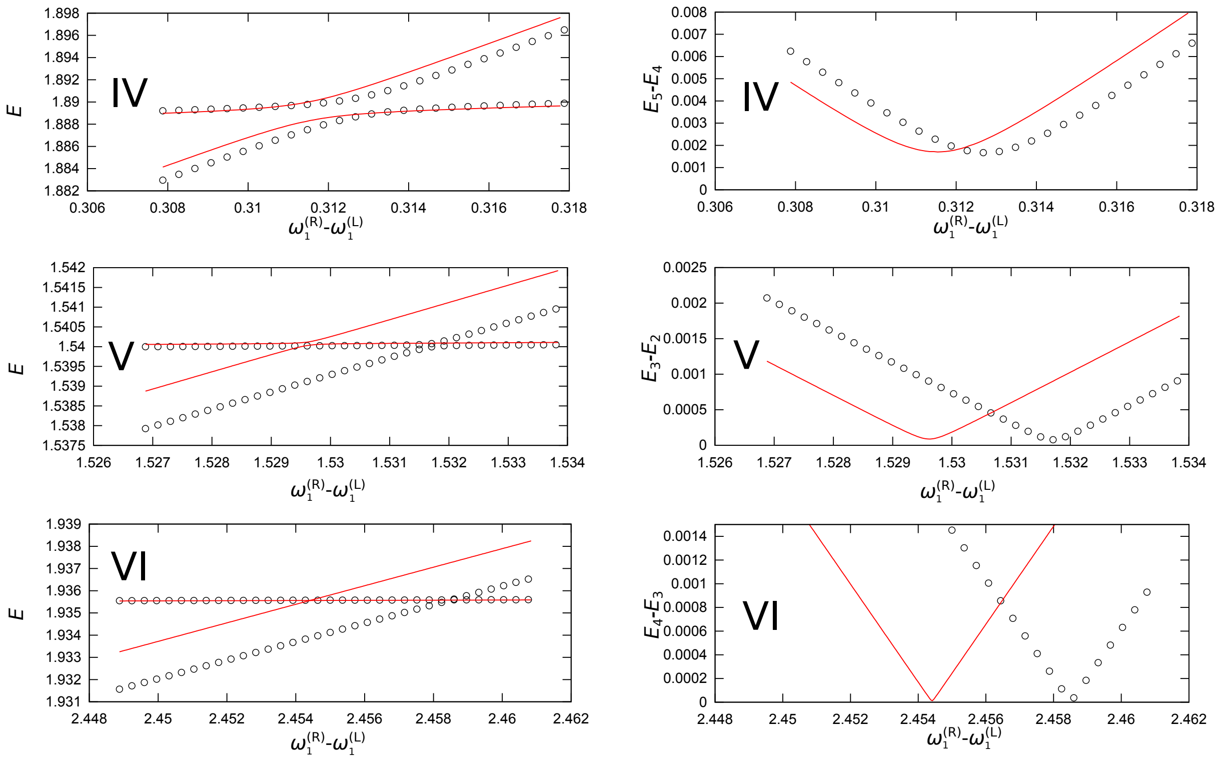

Figure S2: Dependence of vibrational energies (left column panels) and tunneling splittings (right column panels) in the 2D model potential (given by Eq. (10) in the accompanying article) on $\omega_1^{(\mathrm{R})}$ in the region of the avoided crossings, shown in frames IV−VI in Figure S1. Circles represent quantum-mechanical values, while red lines represent values obtained using a combined VCI/instanton approach.

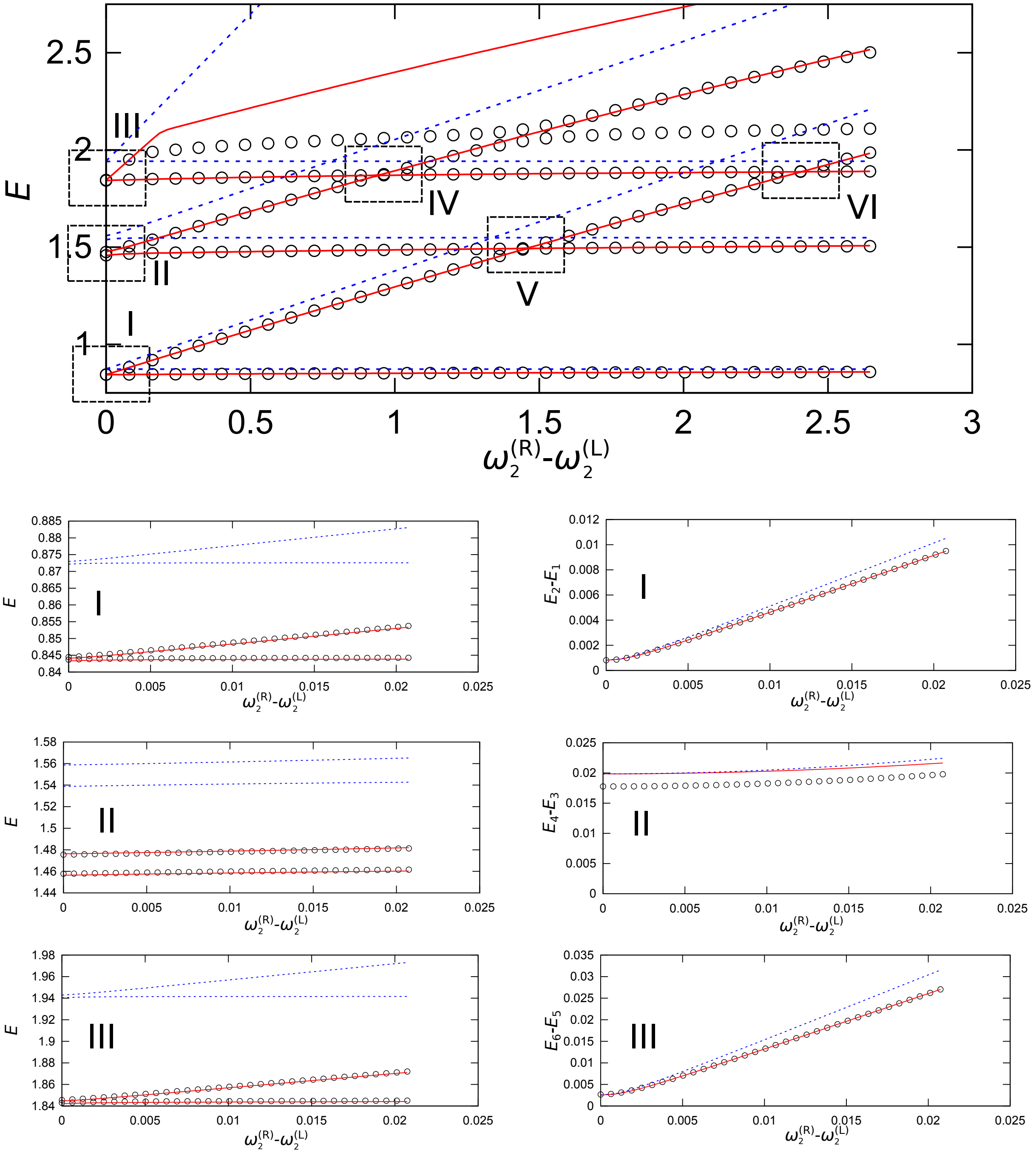

Figure S3: Dependence of vibrational energies of the lowest 6 states in the double-well potential given by Eq. (10) (see accompanying article) on $\omega_2^{(\mathrm{R})}$. Circles represent quantum-mechanical values, blue lines are obtained using instanton method with harmonic energies, red lines are obtained using a combined VCI/instanton approach. Frames I-III in the top panel are shown magnified in the left column panels below, and the dependence of the associated tunneling splittings on $\omega_2^{(\mathrm{R})}$ in the right column panels below.

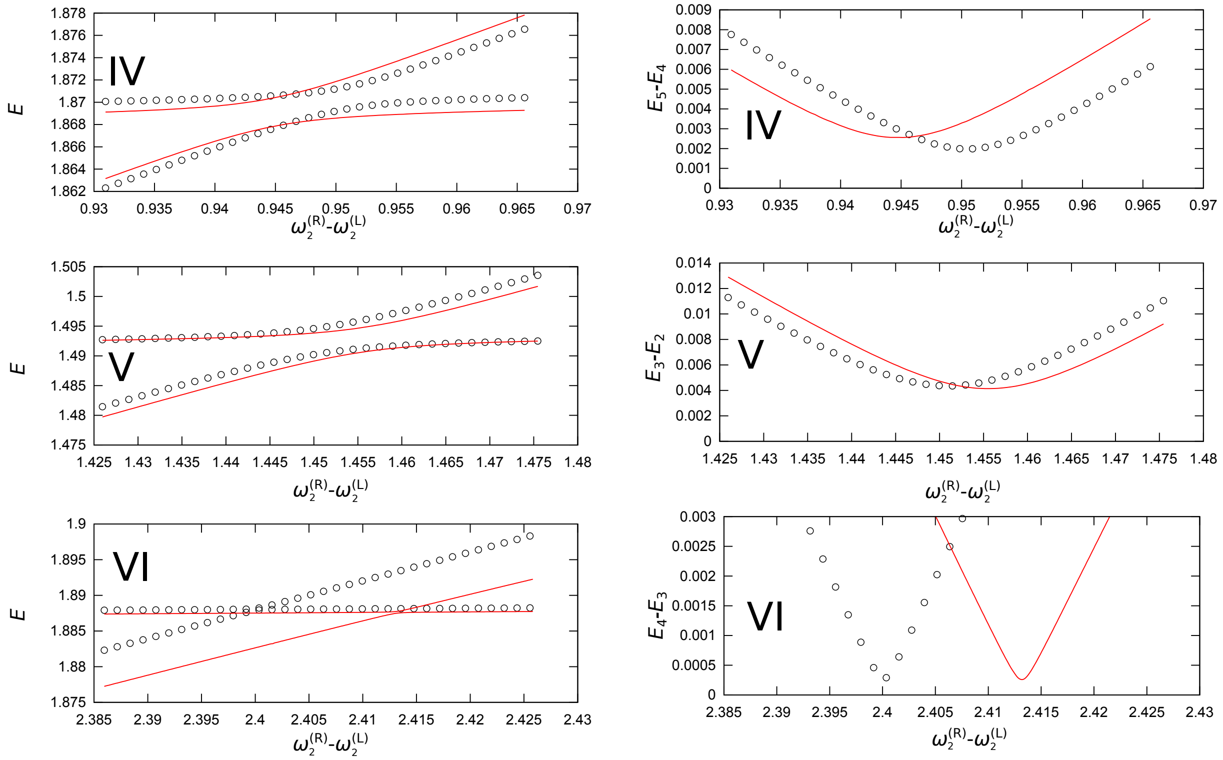

Figure S4: Dependence of vibrational energies (left column panels) and tunneling splittings (right column panels) in the 2D model potential (given by Eq. (10) in the accompanying article) on $\omega_2^{(\mathrm{R})}$ in the region of the avoided crossings, shown in frames IV−VI in Figure S3. Circles represent quantum-mechanical values, while red lines represent values obtained using a combined VCI/instanton approach.

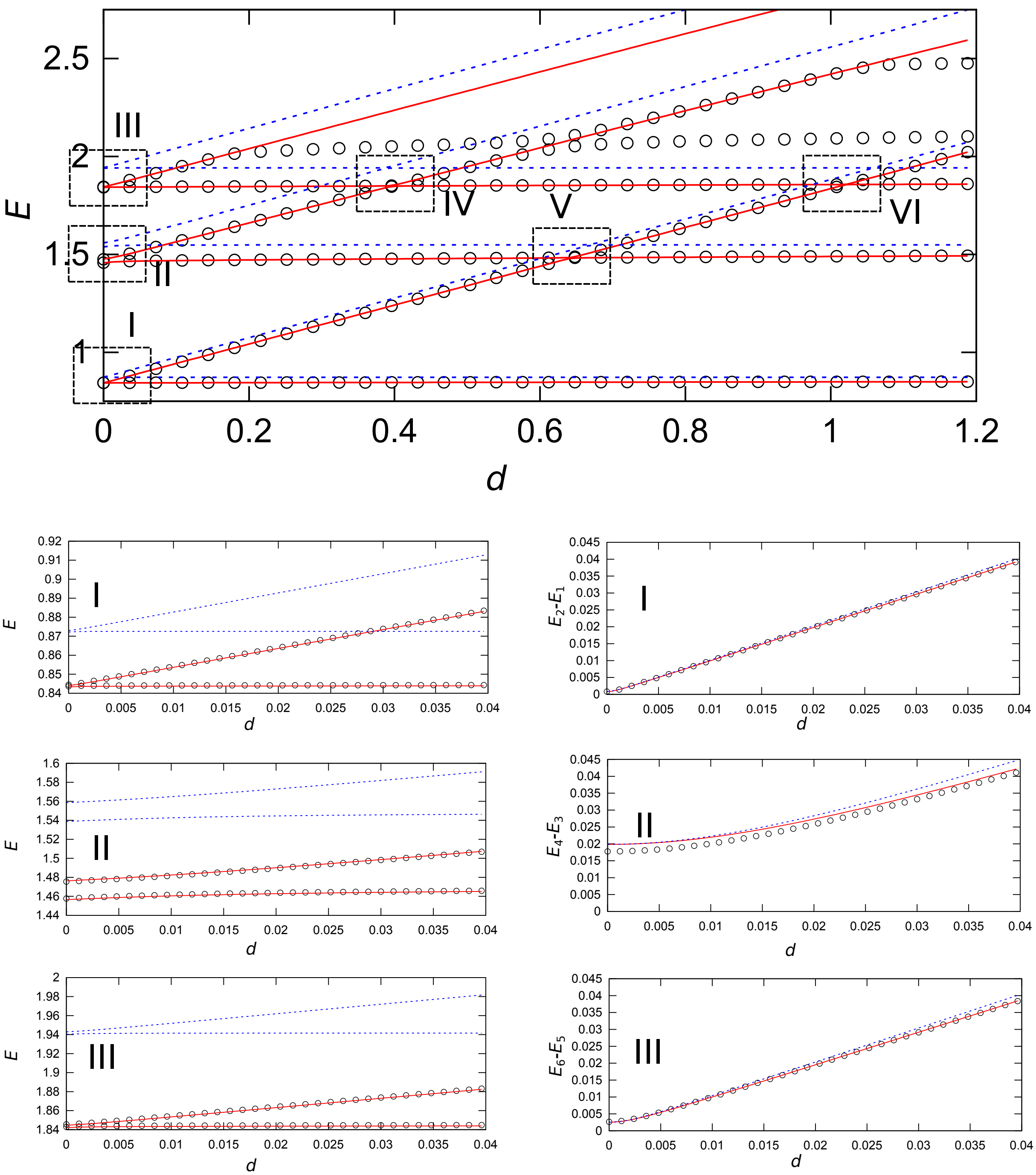

Figure S5: Dependence of vibrational energies of the lowest 6 states in the double-well potential given by Eq. (10) (see accompanying article) on d . Circles represent quantum-mechanical values, blue lines are obtained using instanton method with harmonic energies, red lines are obtained using a combined VCI/instanton approach. Frames I-III in the top panel are shown magnified in the left column panels below, and the dependence of the associated tunneling splittings on d in the right column panels below.

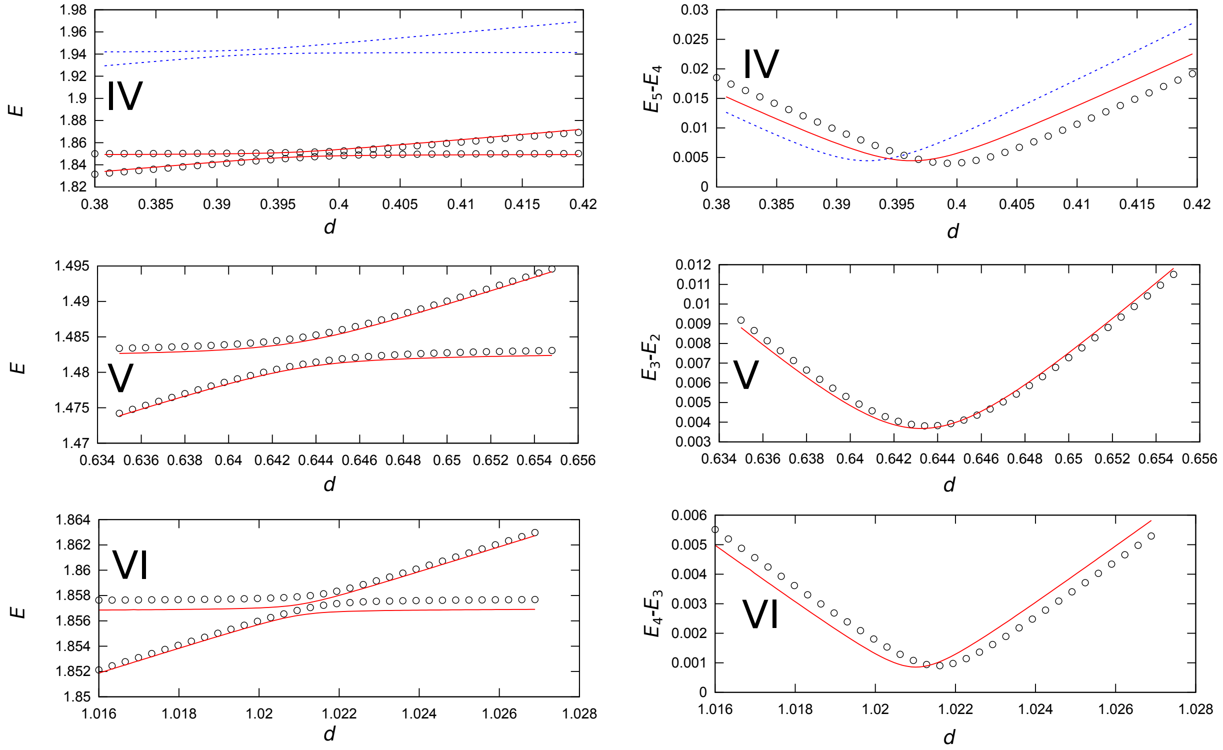

Figure S6: Dependence of vibrational energies (left column panels) and tunneling splittings (right column panels) in the 2D model potential (given by Eq. (10) in the accompanying article) on d in the region of the avoided crossings, shown in frames IV−VI in Figure S5. Circles represent quantum-mechanical values, blue lines are obtained using instanton method with harmonic energies, while red lines represent values obtained using a combined VCI/instanton approach.

References

(S1) Bowman, J. M.; Carter, S.; Huang, X. MULTIMODE: A code to calculate rovibrational energies of polyatomic molecules. *Int. Rev. Phys. Chem.* **2003**, *22*, 533–549.

(S2) Rauhut, G. Efficient calculation of potential energy surfaces for the generation of vibrational wave functions. *J. Chem. Phys.* **2004**, *121*, 9313–9322.

(S3) Carter, S.; Bowman, J. M.; Handy, N. C. Extensions and tests of "multimode": a code to obtain accurate vibration/rotation energies of many-mode molecules. *Theor. Chim. Acta* **1998**, *100*, 191–198.

(S4) Christoffel, K. M.; Bowman, J. M. Investigations of self-consistent field, scf ci and virtual stateconfiguration interaction vibrational energies for a model three-mode system. *Chem. Phys. Lett.* **1982**, *85*, 220–224.

(S5) Bowman, J. M.; Christoffel, K.; Tobin, F. Application of SCF-SI theory to vibrational motion in polyatomic molecules. *J. Phys. Chem.* **1979**, *83*, 905–912.
